# Global distribution and diversity of *Chaetoceros* (Bacillariophyta, Mediophyceae): integration of classical and novel strategies

**DOI:** 10.7717/peerj.7410

**Published:** 2019-08-19

**Authors:** Daniele De Luca, Wiebe H.C.F. Kooistra, Diana Sarno, Chetan C. Gaonkar, Roberta Piredda

**Affiliations:** 1 Department of Integrative Marine Ecology, Stazione Zoologica Anton Dohrn, Napoli, Italy; 2 Department of Oceanography, Texas A&M University, College Station, TX, United States of America

**Keywords:** Biodiversity, Biogeography, Biological records, Chaetoceros, Global distribution, Marine diatoms, Metabarcoding, 18S rDNA, OSD, TARA

## Abstract

Information on taxa distribution is a prerequisite for many research fields, and biological records are a major source of data contributing to biogeographic studies. The Global Biodiversity Information Facility (GBIF) and the Ocean Biogeographic Information System (OBIS) are important infrastructures facilitating free and open access to classical biological data from several sources in both temporal and spatial scales. Over the last ten years, high throughput sequencing (HTS) metabarcoding data have become available, which constitute a great source of detailed occurrence data. Among the global sampling projects that have contributed to such data are Tara Oceans and the Ocean Sampling Day (OSD). Integration of classical and metabarcoding data may aid a more comprehensive assessment of the geographic range of species, especially of microscopic ones such as protists. Rare, small and cryptic species are often ignored in surveys or mis-assigned with the classical approaches. Here we show how integration of data from various sources can contribute to insight in the biogeography and diversity at the genus- and species-level using *Chaetoceros* as study system*,* one of the most diverse and abundant genera among marine planktonic diatoms. *Chaetoceros* records were extracted from GBIF and OBIS and literature data were collected by means of a Google Scholar search. *Chaetoceros* references barcodes where mapped against the metabarcode datasets of Tara Oceans (210 sites) and OSD (144 sites). We compared the resolution of different data sources in determining the global distribution of the genus and provided examples, at the species level, of detection of cryptic species, endemism and cosmopolitan or restricted distributions. Our results highlighted at genus level a comparable picture from the different sources but a more complete assessment when data were integrated. Both the importance of the integration but also the challenges related to it were illustrated. *Chaetoceros* data collected in this study are organised and available in the form of tables and maps, providing a powerful tool and a baseline for further research in e.g., ecology, conservation and evolutionary biology.

## Introduction

Primary biodiversity data can be defined as the basic attributes of observations or records of the occurrence of species (Anderson et al., 2016). For centuries, primary species-occurrence data were mostly obtained from taxonomic descriptions of specimens stored in museums, herbaria and private collections (Chapman, 2005). In the last few years, biological recording has evolved, particularly due to the involvement of citizens and the application of molecular tools (Isaac & Pocock, 2015; Pocock et al., 2015). Indeed, nowadays data are also gathered through satellite tracking and direct or remote observation (Croxall, Briggs & Prince, 1993), frozen tissue collections and seed banks (Chapman, 2005), environmental DNA (August et al., 2015), and citizen science initiatives (Devictor, Whittaker & Beltrame, 2010; Hochachka et al., 2012).

Regardless of their source, data for biological recording are generally presence-only records (opportunistic incidence records, Peterson et al., 2011) since they do not report any info about species absence in an area at the time of the survey. Furthermore, they are subject to bias in space and time, such as uneven sampling due to bias towards easily accessible areas, agreeable weather conditions (Kéry, Gardner & Monnerat, 2010; Isaac & Pocock, 2015), as well as biases in the distribution of economic resources for research, researchers and research effort (Droege, Cyr & Larivée, 1998).

Biodiversity data of planktonic species are traditionally gathered through samples collected once though opportunity, or over time and then either at single sites at long term ecological research (LTER) stations sampled recurrently (e.g., Helgoland Roads, MareChiara; Blanes Bay Microbial Observatory, Hawaii Ocean Time series), or at a string of sites each sampled only once (e.g., Challenger Expedition). A shortcoming of such sampling schemes is that they provide incomplete distribution maps of species with many “blank” regions and seasons. Sampling intensity is often skewed towards areas known to be diverse for taxa of interest because those areas attract the collectors (Prendergast et al., 1993). Examples of initiatives to overcome these issues in the plankton constitute the Sir Alister Hardy Foundation for Ocean Science (SAHFOS) program of putting plankton recorders behind ships to sample tracks recurrently (Southward et al., 2005), and the involvement of the public in citizen science initiatives (Castilla et al., 2015; Busch et al., 2016). The results are usually available in form of taxonomic monographs, checklists, or species descriptions.

The growth of biological records in recent decades led to the establishment of recording protocols and the organisation and storing of such data in freely accessible online portals, such as the Global Biodiversity Information Facility (GBIF; http://www.gbif.org/) (Isaac & Pocock, 2015; Powney & Isaac, 2015) and the Ocean Biogeographic Information System (OBIS; http://iobis.org/). GBIF contains occurrence data for both aquatic and terrestrial species gathered from different sources as natural history collections, environmental monitoring programmes, recording initiatives and citizen scientist projects. Instead, OBIS only focuses on marine biodiversity and biogeographic data but uses the same data sources as GBIF except for museum specimens and herbarium collections. Both contain records that are processed according to the Darwin Core Standard (DwC, Wieczorek et al., 2012), though differences in updating procedures can cause temporary differences in results. Specific for algae is AlgaeBase (Guiry & Guiry, 2018), a repository of information with updated taxonomic info, images, bibliographic items and distributional records of algae curated by phycologists. It focuses mainly on taxonomy, but provides also taxonomically reliable literature sources on distribution.

In recent years, the way biodiversity data are gathered has been revolutionised by the introduction of molecular approaches in taxonomy (August et al., 2015; Lawson Handley, 2015). Taxonomic assignment of specimens based on morphology alone can be inaccurate due to cryptic diversity and intraspecific morphological variation. This is why species identification is often done nowadays using DNA-based methods (Vanormelingen & Souffreau, 2010; Zimmermann et al., 2015). In addition, high throughput sequencing of taxonomically discriminative barcode regions (HTS metabarcoding) has revolutionised our capacity to gather biodiversity data from environmental samples allowing identification of the plethora of species present in complex sample matrices and from mass collections of specimens.

HTS metabarcoding is commonly applied to marine microbial communities, as shown by several recent projects aimed at characterising the diversity and distribution of sea life. Examples are BioMarKs (http://www.biomarks.eu), the Cariaco Microbial Observatory (Edgcomb et al., 2011), Tara Oceans (https://oceans.taraexpeditions.org/en/m/about-tara/), Ocean Sampling Day, OSD (https://www.microb3.eu/osd.html), and time-series at aforementioned LTER stations. These initiatives are in many ways complementary and additive. For instance, Tara Oceans samples have been taken along a global oceanic trajectory on different dates, and the 18S rDNA-V9 region was used as metabarcode (e.g., Malviya et al., 2016), whereas OSD sampled globally as well, but at coastal sites, on a single day (the June 21st solstice) and used the 18S rDNA-V4 region (e.g., Kopf et al., 2015). Tara Oceans and OSD constitute a valuable resource for biological recording and provide information from areas difficult to access (Ji et al., 2013). Their standardised procedures, including a centralised hub for laboratory work and data processing guaranteed consistency and data interoperability, and the resulting sequences and contextual data are now publicly available. Previous examples of the use of OSD or Tara Oceans datasets to map phytoplankton distribution were performed using only one of two datasets, without integration of classical sources and at high taxonomic levels (e.g., Malviya et al., 2016; Lopes dos Santos et al., 2017; Penna et al., 2017; Tragin & Vaulot, 2018).

As result of all these metabarcoding activities, a wealth of different kinds of plankton biodiversity data is now available from various sources and in different formats, waiting to be applied to research questions in biogeography, biodiversity estimations, conservation and climate change biology. The integration of all these classical data sources and results from HTS metabarcoding may help improving environmental monitoring, -management and -policy decisions (Kelly et al., 2014; Thomsen & Willerslev, 2015).

In this paper, we highlight the importance of the integration of classical and novel primary biodiversity data as well as the challenges related to it through the assessment of the global distribution of *Chaetoceros*. *Chaetoceros* is a highly diverse genus of marine planktonic diatoms (VanLandingham, 1968; Rines & Hargraves, 1988), and an abundant one globally (Leblanc et al., 2012). Genetic distances across its diversity (e.g., Gaonkar et al., 2018) are comparable to those observed among higher taxonomic categories (e.g., families or even orders) in other diatom lineages. Cryptic diversity seems to be extensive in this group (Kooistra et al., 2010; Balzano et al., 2017; Gaonkar et al., 2017; Li et al., 2017) affecting the mapping of species distribution patterns based on morphological data.

We first explore the potential of different sources of occurrence data at assessing distribution and abundance of a highly diverse phytoplankton genus as well as its species richness in various regions all over the world. Then we assess distribution patterns of *Chaetoceros* species using metabarcoding data and compare them with literature data in selected species in order to evaluate their potential and limits in biodiversity assessments.

## Materials & Methods

### Data collected from available public repositories, literature and checklists

**Figure 1:**
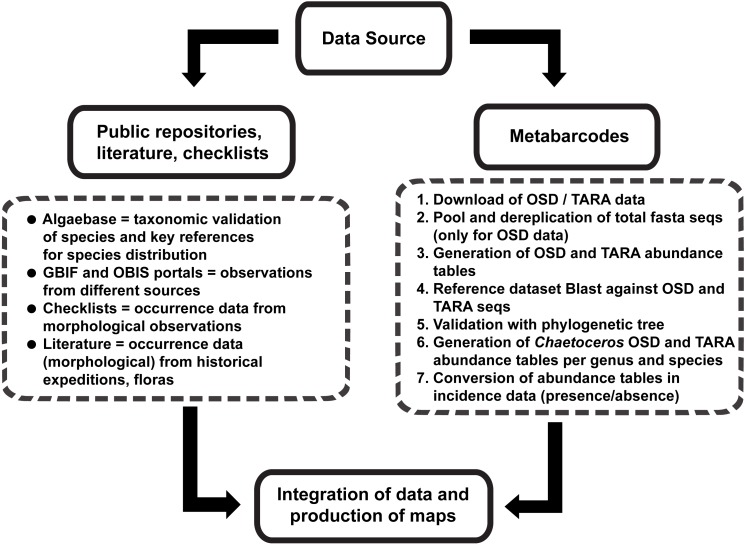
Graphical representation of the main workflow.

In order to collect comprehensive info about the distribution of *Chaetoceros* species, we developed a pipeline that is summed up in Fig. 1. We started our search consulting AlgaeBase (Guiry & Guiry, 2018). Upon typing “*Chaetoceros*” in the field ‘search genus’, we performed a preliminary filtering, taking into the account only taxonomically accepted species. For these, we retrieved the listed literature to record occurrences. In parallel, we searched Google Scholar for main checklists and distributional records in the literature using as keywords “*Chaetoceros*/phytoplankton distribution”, “…checklist”, “…occurrence” and “…biogeography”. Papers resulting from cited literature were also considered.

We consulted all the gathered papers containing info at the species-level but considered for the analyses only the ones focused on phytoplankton taxonomy and whose species names were accepted in Algaebase. For additional sources of occurrence data at the genus level, we checked GBIF and OBIS, using the query “Chaetoceros”, and downloaded the resulting occurrence data using the R (RCoreTeam, 2018) working packages “rgbif” (Chamberlain, 2017) and “robis” (Provoost & Bosch, 2018) for GBIF and OBIS, respectively. Data were plotted using the packages “maps” (Becker et al., 2018) and “ggplot2” (Wickham, 2016). A list of abbreviations of the main datasets utilised in the present study is provided in Table 1.

### Data generated from molecular sources

We used the V4-18S metabarcodes from OSD and the V9-18S metabarcodes from Tara Oceans to assess the distribution of *Chaetoceros* globally. For the OSD dataset, we downloaded the V4 lgc workable data (e.g., data already pre-processed in order to derive common data sets on which to base follow-up analysis) available at the website https://mb3is.megx.net/osd-files?path=/2014/datasets/workable. Details of sampling protocols and types of molecular data generated are available at https://github.com/MicroB3-IS/osd-analysis/wiki/Guide-to-OSD-2014-data, whilst details of pre-processing can be found at https://github.com/MicroB3-IS/osd-analysis/wiki/Sequence-Data-Pre-Processing. The workable fasta files, downloaded for each of 144 geographical sampling sites, were pooled and a total fasta file generated, containing the non-redundant (unique) sequences and a table containing their distribution along the sites (Total OSD abundance table) using mothur v1.41.1 (Schloss et al., 2009). For the Tara Oceans dataset, we downloaded the V9-metabarcode dataset (De Vargas & Audic, 2017) available at https://doi.pangaea.de/10.1594/PANGAEA.873277 and at ENA (accession number: PRJEB9737) and, following the same pipeline described above, from the total 210 sampling sites we generated a total unique fasta file and a Total Tara Oceans abundance table.

**Table 1:** List of abbreviations of the datasets utilised in the present study.

GBIF	Global Biodiversity Information Facility
OBIS	Ocean Biogeographic Information System
OSD	Ocean Sampling Day

To generate distribution data, we used a selection of the taxonomic validated *Chaetoceros* 18S rDNA sequences (Gaonkar et al., 2018). In particular, the reference barcode dataset included 202 *Chaetoceros*, 15 *Bacteriastrum* and 29 outgroup taxa. The V4 and V9 fragments were extracted from the full-length 18S genes and aligned using MAFFT online (Katoh, Rozewicki & Yamada, 2017). In order to avoid mis-assignations at the species level, for the two fragments (V4 and V9) we simulated several thresholds of clustering based on genetic distances (commands “dist.seqs” and “cluster” in mothur) (Schloss et al., 2009).

The V4 and the V9 reference sequences were used as queries for a local blast against the OSD and Tara Oceans datasets. For the mapping at genus level, we set the threshold at 90% of similarity and from the outputs of BLAST we retained only the metabarcode hits having a query coverage with the reference >370 bp in the analysis of V4 OSD dataset, and >105 bp for V9 Tara Oceans dataset. The metabarcodes extracted were aligned with the references, including outgroup taxa, using MAFFT online (Katoh, Rozewicki & Yamada, 2017) and two phylogenetic trees were then built in FastTree v2.1.8 (Price, Dehal & Arkin, 2010), using the GTR model, and visualised in Archaeopteryx v0.9901 (Han & Zmasek, 2009). Metabarcode hits clustering within the outgroup clades were excluded, and the remainder considered as validated *Chaetoceros*. Their abundances and distributions were extracted from the Total OSD and Tara Oceans abundance tables to generate the *Chaetoceros*-genus OSD abundance table (Table S1) and *Chaetoceros*-genus Tara Oceans abundance table (Table S2). For the mapping at species level, we first evaluated the results from the analyses described above for the V4 and V9 fragments (calculation of the genetic distances and simulation of several thresholds of clustering). Based on these, we extracted only the blast hits assigned in the range 100–99% of similarity. This range was identified as the best compromise between the precision required to an assignation at species level and the intra-species variation that could occur especially at global level. After the blast, we applied the same procedure described above for the genus level (alignment and generation of tree) to validate the assignations and we generated the *Chaetoceros*-species abundance table for the OSD (Table S3) and for Tara Oceans (Table S4) datasets.

The *Chaetoceros*-genus abundance tables were used both in term of occurrence and abundance of V4 and V9 reads in each sampling site. Abundance values were log10-transformed and plotted using *ggplot2* (Wickham, 2016).

Finally, to explore in detail the performances of classical and molecular data, we selected three species as case studies: (i) *C. tenuissimus* as an example of a cosmopolitan species; (ii) *C. gelidus* as an example of a species with a restricted distribution; and (iii) *C. neogracilis* as an example of a putative cryptic species complex.

## Results

### Data collected from available public repositories, literature and checklists

According to AlgaeBase, the genus *Chaetoceros* contained 370 species names and 172 intraspecific ones, 220 of which have been flagged as taxonomically accepted species based on the available literature (searched on 15/10/2018). This discrepancy is due to the occurrence of many homotypic or heterotypic synonyms in the literature as well as species of uncertain taxonomic status, which need taxonomic revision or validation. We further filtered the 220 taxa flagged as taxonomically accepted (e.g., removing entries occurring twice) obtaining a final table (Table S5) with 173 entries at the date of the search. We considered the latter taxa in the count for species richness from literature data (see below).

**Figure 2:**
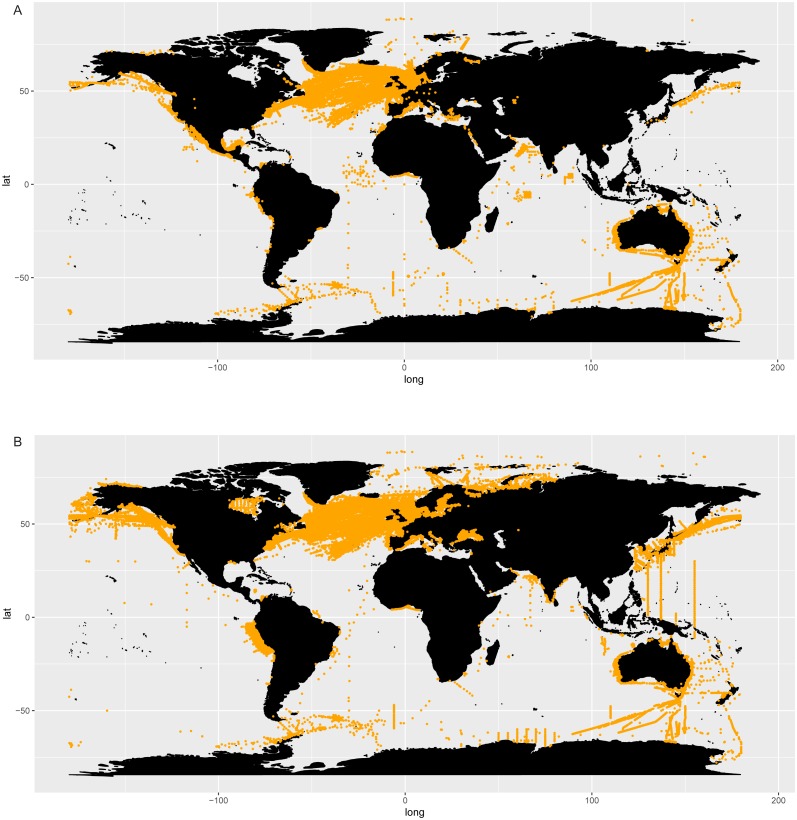
Occurrence of *Chaetoceros* using GBIF (A) and OBIS (B) data.

The distribution map of *Chaetoceros* obtained using GBIF data (Fig. 2A) was based on 201,047 occurrence records from 1863 to 2018 (https://www.gbif.org/occurrence/charts?q=chaetoceros). Data were mostly from human observations (75.7%) and preserved specimens (20.2%) (GBIF.org, 14 September 2018, GBIF Occurrence Download; https://doi.org/10.15468/dl.nofa8w). The definition of records is available at https://gbif.github.io/gbif-api/apidocs/org/gbif/api/vocabulary/BasisOfRecord.html. Filtered occurrence data from GBIF are also available as supplementary info (Table S6). No information from literature was available for *Chaetoceros* in GBIF data. Most of the observations were from the North Atlantic Ocean between 35°−60°N and −80°W −10°E (Continuous Plankton Recorder Dataset, SAHFOS, 83,513 counts; Réseau d’Observation et de Surveillance du Phytoplancton et des Phycotoxines, REPHY, 17,742 counts; QUADRIGE, 12,458 counts), followed by the Pacific coasts of North and Central America and Australia (Fig. 2A).

The distribution map obtained searching *Chaetoceros* in the OBIS database (Fig. 2B) contained 389,206 records from 1863 to 2016 (Table S6). Most of observations were from the World Ocean Database 2009 (119,592), followed by the Continuous Plankton Recorder (86,309) and the Japan Oceanographic Data Center Dataset (JODC, 31,388).

*Chaetoceros* occurrence data were found in 435 GBIF datasets and 179 OBIS datasets, of which 20 were shared (Table S6).

**Figure 3:**
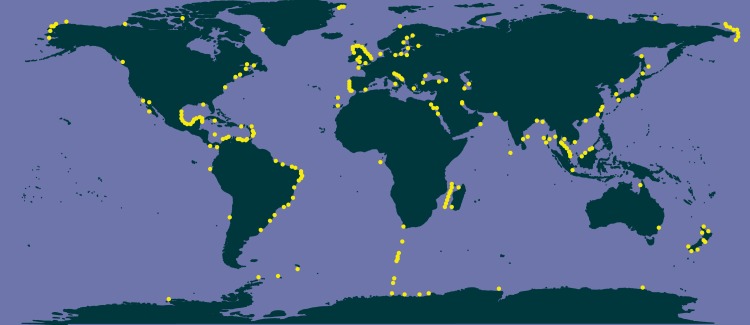
Occurrence of *Chaetoceros* using literature data.

The literature search conducted in Google Scholar resulted in 84 main bibliographic references reporting data of *Chaetoceros* occurrences (Table S7). These data encompassed both single observations and time series across the world, covering a period from 1873 to 2017 (Table S7). None of these bibliographic references (checklists and papers) was contained in GBIF or OBIS datasets (Table S6). According to these data, *Chaetoceros* species occurred everywhere (Fig. 3).

**Figure 4:**
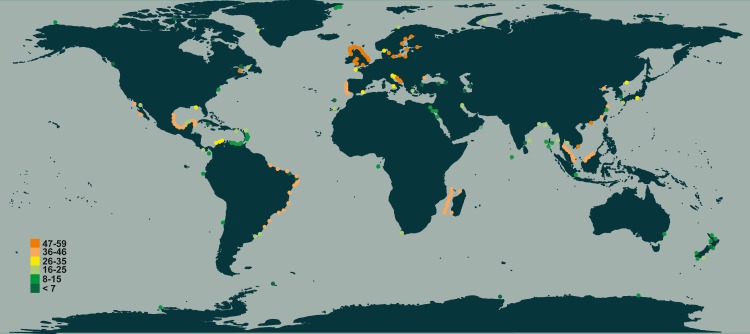
Species richness of *Chaetoceros* estimated from literature data. Colours refer to the different classes of abundance (number of species recorded).

In terms of species richness, here defined as the number of valid species recorded in each locality’s checklist, we found the highest values in temperate European coastal waters (North Sea, Baltic Sea, and middle Adriatic Sea, Fig. 4), followed by the tropical and subtropical waters of Brazil, Mozambique Channel and Indonesia (Fig. 4). The lowest numbers were found in the subpolar waters alongside the coasts of northern countries (Canada, Greenland, Norway and Russia) as well as in the equatorial ones of the southern oceans (Fig. 4).

### Data generated from molecular sources

Based on the generation of distances and simulation of clustering thresholds, the clustering at 100% similarity of the V4 *Chaetoceros* reference dataset (unique or non-redundant sequences) resulted in the collapse of only multiple strains from the same species, whereas the clustering at the 99% similarity threshold resulted in the collapse of 14 species (Table S8). Instead, in the V9 *Chaetoceros* reference dataset clustering at 100% identity already produced the collapse of 17 species, resulting in limitations in the mapping at species level (Table S8).

**Figure 5:**
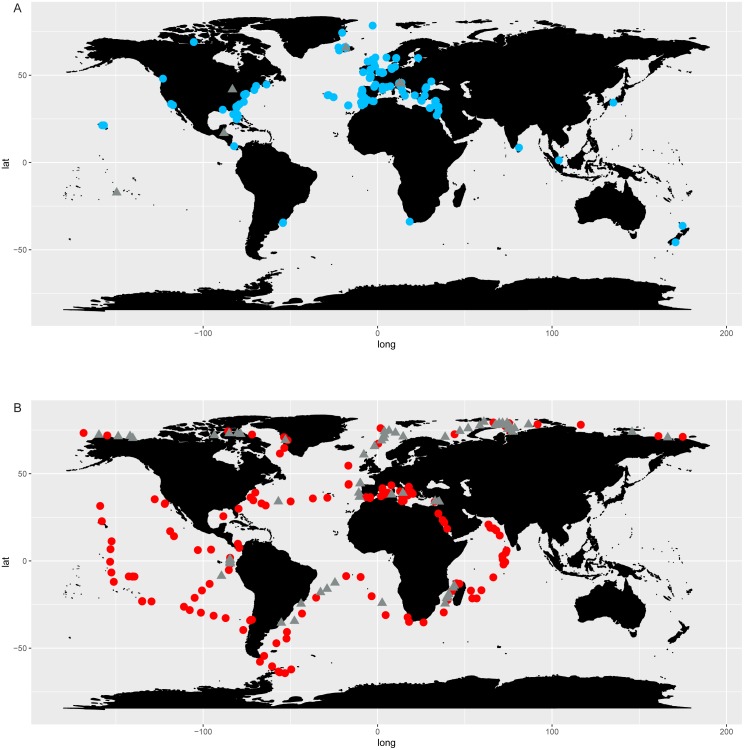
*Chaetoceros* distribution according to OSD (A) and Tara Oceans (B) data. Dots indicate presence of *Chaetoceros* taxa in the sampling stations, whilst triangles their absence.

**Figure 6:**
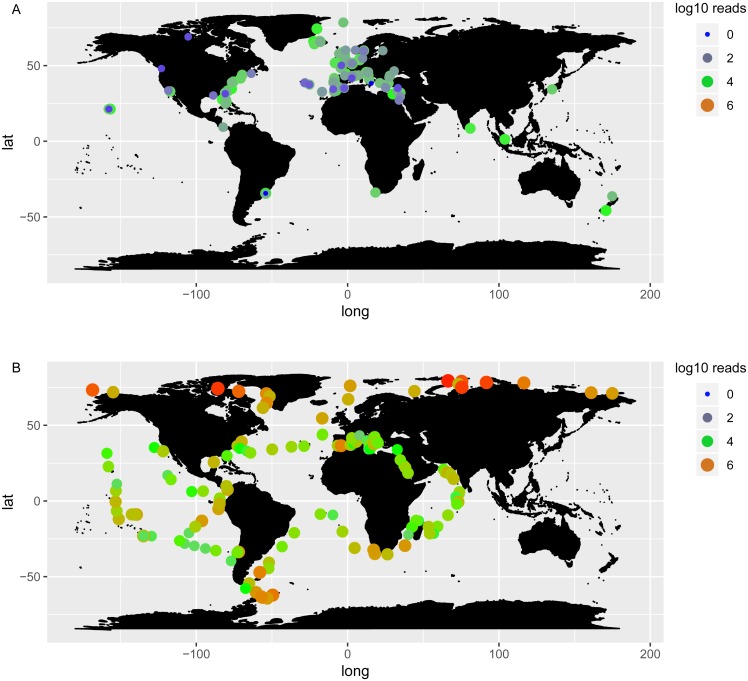
Log10 abundance of *Chaetoceros* reads according to OSD (A) and Tara Oceans (B) datasets. Size and colours of the circles refer to the abundance.

*Chaetoceros* taxa were found in 138 out of 144 OSD sampling sites (96%) and 146 out of 210 Tara Oceans stations (70%), highlighting a wide distribution of the genus (Fig. 5, Table S9). A plot of abundances, both in OSD and in Tara Oceans datasets, showed that *Chaetoceros* was equally abundant in the northern as in the southern hemisphere (Fig. 6). The highest abundances (in terms of reads) were mostly found in the polar to temperate regions of the two hemispheres, with some exceptions in the equatorial coastal waters of India and Indonesia (Fig. 6A). Lowest abundances were encountered in the subtropical to equatorial zones, especially in open ocean stations in the case of Tara Oceans dataset (Fig. 6B), in the Red Sea for both datasets, and other few sites in the OSD dataset (Fig. 6A).

At the species level we generated, at 99% the similarity threshold, a map of occurrence in the OSD and Tara Oceans datasets for each of the 69 *Chaetoceros* species (Article S1, Table S10). The only exceptions were *C.* cf. *vixvisibilis* Na16A3 and *C.* sp. Clade Na28A1 strain Na26C1, in which the collapse of barcodes prevented the plot of occurrences in Tara Oceans stations at species level. Taking into the account that the OSD dots (blue) are heavily skewed towards the coasts of the eastern US and Europe and taken in the beginning of summer and that the Tara Oceans dots are in large part oceanic, some general patterns emerged (in Article S1). Different cryptic species within morpho-species (e.g., *C. brevis, C. curvisetus, C danicus, C. debilis, C. diadema, C. didymus, C. lorenzianus*-complex, *C. peruvianus, C*. cf. *tortissimus*) often showed markedly different global distribution patterns. Several members of the subgenus *Chaetoceros* were found to be predominantly oceanic (e.g., *C*. cf. *pseudodichaeta, C. dichaeta, C. eibenii, C. peruvianus*), though not all of them, and some *Hyalochaete* species were also found to be oceanic (e.g., *C. diadema* 1, *C. debilis* 2 strain MM24-A3, *C. rotosporus*). Other commonly encountered species were clearly coastal (e.g., *C. socialis, Chaetoceros* sp. Clade Na11C3). Certain species can be considered cosmopolitan (e.g., *C. eibenii, C. peruvianus* 1, *C. rostratus, C. rotosporus, C. tenuissimus*) whereas others were restricted to the cold temperate and boreal regions (*C. cinctus, C. constrictus, C. debilis* 1 and 2 (both strains), *C. gelidus, C. neogracilis*) or the warm-temperate to tropical regions (e.g., *C.* cf. *pseudodichaeta, C.* cf. *tortissimus, C. curvisetus* 3, *Chaetoceros* sp. Clade Va7D2). Despite the heavy skew of OSD data to the North Atlantic, a few species seemed confined to a particular region, showing many dots there, whereas they were not observed in other regions (*C. affinis, C. debilis* 2 strain L38-A2).

**Figure 7:**
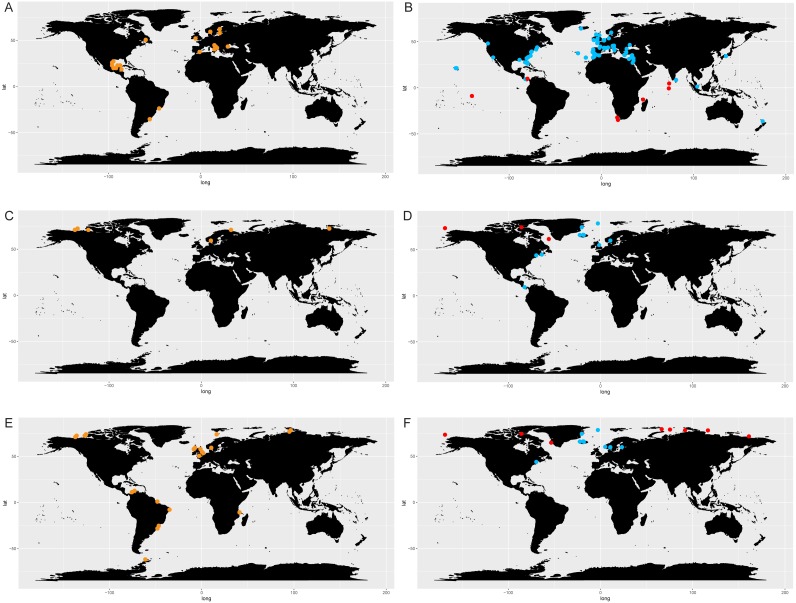
Distribution of *C. tenuissimus* (A, B), *C. gelidus* (C, D) and *C. neogracilis* (E, F) according to literature (orange dots) and metabarcoding data (yellow dots). Maps containing the sites considered for literature and metabarcoding data are found in Fig. 3 and Article S1 respectively.

The comparison of literature and genetic (metabarcoding) data in selected species of *Chaetoceros* (Fig. 7) showed consistency in the signal for *C. tenuissimus* and *C. gelidus*, and highlighted the discrepancy between the morphological and molecular data *C. neogracilis*.

Literature (Fig. 7A) and metabarcoding data (Fig. 7B) confirmed a cosmopolitan distribution of *C. tenuissimus*, with metabarcoding data providing new records for African, Asian and New Zealand coasts (Fig. 7B).

For *C. gelidus*, genetic data from OSD and Tara Oceans (Fig. 7D) confirmed the distribution area of literature data (field observations, Fig. 7C) but also included new records for Canada, North Scotland and Iceland (Fig. 7D). The species was also found in one OSD station in the Caribbean side of Panama coasts, but at very low abundance (2 reads at 100% similarity).

According to literature, *C. neogracilis* was found both in the northern and southern hemisphere (Fig. 7E). On the contrary, occurrence data from metabarcoding revealed instead a distribution limited to the northern hemisphere, so covering just a small part of the distribution range known from literature data (Fig. 7F).

## Discussion

The wealth of environmental -omics data gathered at many different locations in recent years calls for their combined reuse to address questions in biogeography, ecology, conservation and evolutionary biology. Results can be compared with what is known from classical sources of information, gathered over much longer time windows. Such combined studies could lead to a far better understanding of especially the protistan diversity (Troudet et al., 2017). In this paper, through the assessment of the global distribution of *Chaetoceros* using metabarcoding and morphological sources, we highlight both the importance of the integration of data and the challenges one may encounter when integrating such distinct types of data.

### General considerations

Of all the non-molecular data, the most complete picture of *Chaetoceros* distribution was provided by the GBIF and OBIS platforms, which contain a huge amount of data from different sources (fossils, literature, machine and human observations, museum and herbarium specimens) and cover a wide time scale (in this case more than 150 years). Through the two portals, a huge amount of data is easily accessible and searchable showing the importance of organising biodiversity data in way that facilitate their access.

In spite of the fact that OBIS is a resource dedicated to marine organisms already included in GBIF database, we did not recover the same number of records and datasets from the two sources. Differences in updating data procedures can cause temporary differences in results; besides, some kinds of information, as museum collections, are only available in GBIF, demonstrating the necessity to interrogate both databases also in the case of marine species to strive towards a more complete mapping.

The overview provided by the Google Scholar search of the main phytoplankton checklists is, despite the obvious limitations, able to provide the main distributional areas of the genus. Google Scholar can be considered as a convenient starting place to commence a literature search, not an endpoint. It has among its advantages the fact that is easily accessible to retrieve data stored in libraries’ catalogues and databases going back centuries. Since this approach is highly sensitive to the kind and order of keywords used for the search, we cannot exclude the possibility of having missed some information, even if multiple searches were performed. Nonetheless, we retrieved datasets not included in GBIF or in OBIS. This aspect underlines that despite the big effort to generate and update these global databases, not all information is yet included. Furthermore, it highlights the difficulty for the researches to produce an exhaustive assessment of all the available data of a particular taxon.

The two global metabarcoding datasets OSD and Tara Oceans, despite being biased in space and time, provide an overall distribution map of the genus that is comparable to the one obtained from GBIF and OBIS. This clearly highlights that, despite some weaknesses (e.g., Coissac, Riaz & Puillandre, 2012; Ficetola et al., 2015), the information available in metabarcode data, in less than a decade from its beginnings, is comparable with classical morphological records gathered over hundreds of years. Metabarcoding can be considered a powerful complement rather than a substitute of other data sources (Bush et al., 2017). It is not ready yet to replace the classical methods of biodiversity recording because the downstream bioinformatics procedures to sort out the species still need work. For instance different % similarity thresholds for clustering metabarcodes into taxonomic units lead to radically different numbers of species. Moreover, inferences at the species level using metabarcoding data need taxonomically validated reference sequences able to provide unambiguous species identifications. Nonetheless, metabarcode data already add massively to our knowledge of species distribution. For instance, the Tara Oceans dataset added new occurrence information for equatorial regions and other open ocean sites in the southern hemisphere, contributing to our knowledge in these still poorly investigated areas.

At the moment, molecular and classical sources tend to be curated and stored in separated repositories or infrastructures, forcing users interested in integration of such sources to do a non-trivial trawl across these sources of data using a variety of analytical procedures. To our knowledge, the only global effort addressing this matter is a recent cooperation between GBIF and EMBL-EBI with the aim at integrating metagenomics data from EMBL-EBI infrastructure and the species occurrence into GBIF (https://www.gbif.org/news/6ewyUhBpRYammYWI2CgsM4/biodiversity-infrastructures-to-crosslink-metagenomics-and-species-occurrence-data). Certainly, molecular approaches can improve our knowledge, reducing mis-assignments into the wrong species or lumping into cryptic species complexes, and aiding accurate identification of rare, small and morphologically featureless species. However, there are also limits to what can be achieved with metabarcodes, especially with short fragments used in metabarcoding, closely related species may have identical regions affecting their discrimination at species level (Cowart et al., 2015; Mordret et al., 2018; Piredda et al., 2018).

### Global distribution of *Chaetoceros*

Our results show that all data sources (GBIF, OBIS, Google Scholar search, OSD and Tara Oceans) support a cosmopolitan distribution of the genus *Chaetoceros* as suggested by Rines & Hargraves (1988) using only classical sources, and Malviya et al. (2016) using only metabarcoding data. In terms of occurrence, *Chaetoceros* taxa showed a global distribution ranging from coastal areas to open ocean and from polar to tropical regions. However, the different data sources point out a prevalence of occurrences in the temperate coastal waters between the temperate waters 60°N and 30°N and in the subtropical and equatorial ones between 30°N and 30°S. This can be due to the presence in such regions of various habitats (upwelling zones, lagoons, oligotrophic as well as eutrophic regions) and the marked seasonality in the water, which offer opportunities of co-existence of species through spatial or seasonal niche partitioning. Boreal regions are poorer probably because there is only the single summer season for phytoplankton growth.

With some exceptions (e.g., Hernández-Becerril & Granados, 1998) for the Gulf of Mexico and (Hernández-Becerril, 1996) for the Mexican Pacific, the tropics are generally under-investigated for species diversity, though this is now ameliorated by recent studies in those regions (Li, Lundholm & Moestrup, 2013; Li et al., 2017; Chamnansinp, Moestrup & Lundholm, 2015).

In general, patterns of abundance in both molecular datasets suggest that *Chaetoceros* is equally abundant in the northern as in the southern hemispheres, with a paucity of reads from many sites located in the open ocean. However, evaluation of geographic range is strongly affected by the base of knowledge we use. A previous mapping of *Chaetoceros* using the Tara Oceans dataset by Malviya et al. (2016) used only 46 stations. In the latter study, *Chaetoceros* was found to be highly abundant in the Southern Ocean and absent in the polar regions of the northern hemisphere. Our analysis, using the complete Tara Oceans dataset (210 stations), showed that *Chaetoceros* is present also in the polar regions of the northern hemisphere, highlighting the fact that the wider the coverage of sampling and/or the integration with data from other sources, the better the resolution of distribution.

The direct comparison of literature and metabarcoding data in three selected species of *Chaetoceros* shows the power of novel molecular data coupled with classical occurrence data.

In the case of *C. tenuissimus,* the molecular data allowed to increase the geographic range of distribution of this cosmopolitan species with new records in African, Asian and New Zealand coasts. In the case of *C. gelidus,* a close relative and look-alike of *C. socialis,* molecular data confirmed our previous knowledge on its restricted distribution in cold water, also adding new records for Canada, North Scotland and Iceland. For this reason, we interpret the occurrence of two reads found in one OSD station along the Caribbean coasts as a glitch rather than a record, though it could represent a closely related tropical species. Recently, Gaonkar et al. (2017) uncovered two additional cryptic species in what in light microscopy can all be considered *C. socialis sensu lato*. However, global changes could alter limits both in cosmopolitan or restricted species with consequent range expansion or contraction, highlighting the importance to generate baseline studies of the geographic distribution range of taxa to use as bases for future comparisons.

More complex is, instead, the case of *C. neogracilis*. The epithet *C. neogracilis* (*C. gracile* Schütt) has been attributed in the past to many small, unicellular *Chaetoceros* taxa reported worldwide (Rines & Hargraves, 1988). A recent study by Balzano et al. (2017) conducted in the Beaufort Sea (Canadian Arctic) revealed the occurrence of morphologically similar strains sharing identical 18S rDNA sequences, but belonging to four distinct clades based on 28S rDNA, ITS-1 and ITS-2 markers. It is beyond the purpose of this paper to argue if they belong to the same biological species or not, but since OSD and Tara Oceans datasets are based on the 18S gene, we regarded these entities as one single species. The reference barcode from Balzano et al. (2017) blasted against the two datasets found identical sequences only in the cold waters of the northern hemisphere strongly suggesting that *C. neogracilis* is a species restricted to polar regions of the northern hemisphere (as highlighted also by Balzano et al. (2017).

The maps of occurrences generated using the OSD and Tara Oceans datasets for each of the 69 *Chaetoceros* species, confirm some of the existing ideas about their distribution patterns from the literature, for instance the oceanic distribution of many species in the subgenus *Chaetoceros*, the cold temperate-boreal nature of some species and the more warm-temperate to tropical distribution of others. Yet, the maps also provide new insights on biogeography of marine diatoms; the distinct distribution patterns of the various cryptic species within morpho-species suggest that these species, despite their highly similar morphology, fulfil different roles in the global marine ecosystem.

According to available literature, few endemic diatom species are known, and they are mostly freshwater (e.g., *Eunophora* in Tasmania and New Zealand, (Vanormelingen, Verleyen & Vyverman, 2008) and *Cyclotella minuta* in Lake Baikal, (Mackay et al., 2006)) or from saline inland lakes (e.g., *Aulacoseira baicalensis*). Claims of putative endemic marine diatoms exist (e.g., Percopo et al., 2016) and are discussed in Mann & Vanormelingen (2013). In the specific case of *Chaetoceros*, Hernández-Becerril (1996) recognised that little efforts have been made to assess its world distribution but, starting from literature data available and personal observations, he grouped taxa according to major regions as inhabitants of cold waters, temperate to subtropical waters, world-wide warm waters and tropical and subtropical waters. Our metabarcoding data suggest that cases of endemism or restricted geographical distributions can also be found in the marine environment as highlighted for species whose occurrence seems limited to single basins as the Mediterranean Sea (*C. diversus* 1) or part of it (*C. throndsenii* in the Adriatic Sea) as well as distribution restricted to climatic zones (e.g., the polar to temperate zones for *C. constrictus*, *C. danicus* strain RCC2565, *C. debilis* 1 and *C. neogracilis*).

## Conclusions

The knowledge of the geographic range of species is a key issue in ecology, conservation and evolutionary biology, allowing investigating causes and consequences of their limits. Climate change can alter these limits with consequent range expansion or contraction, and several examples have been reported (Walther et al., 2002; Parmesan & Yohe, 2003; McLachlan, Clark & Manos, 2005). This process is supposed to be underway, stressing the need to collect, integrate and summarise data available to create a primary biodiversity data baseline. These collections provide bases for future comparisons or model predictions to support biodiversity change assessments. In this study, we highlight both the importance of the integration of data and the challenges related on it, generating a comprehensive primary baseline of the geographic distribution range and diversity for *Chaetoceros,* one of the most diverse and abundant genera of marine planktonic diatoms. In our protocol, we first identified several potential sources for classical data (online databases, literature data) and for molecular data (global surveys as the OSD and Tara Oceans datasets). Then, collected and newly generated data were integrated and organised in maps and tables ready to use and support marine scientists for several purposes, ranging from simple diversity comparison to evolutionary and ecological studies. The outcomes showed that all three kinds of data utilised (online databases, literature and metabarcoding) have more or less the same power of resolution in determining the distribution of the genus, with GBIF and OBIS infrastructures (which include different sources of data) performing slightly better. Data also highlighted that there is no single hotspot of species diversity, and that the highest number of species is found in the coastal temperate waters of the northern hemisphere as well as in the tropics and subtropics of the southern hemisphere, besides the presence of endemism in marine diatoms. Furthermore, *Chaetoceros* is equally abundant in polar to temperate regions of the northern and southern hemispheres. In the case of specific taxa, we have showed that both data are useful to detect cases of cosmopolitan vs. restricted distribution (*C. tenuissimus* and *C. gelidus*), to spot cold-water species that can be used as early sentinels of environmental changes (*C. gelidus* and *C. neogracilis*).

##  Supplemental Information

10.7717/peerj.7410/supp-1 Table S1 Chaetoceros-genus OSD abundance table The raw data of the abundance of V4 reads of *Chaetoceros* in each sampling locality.

10.7717/peerj.7410/supp-2 Table S2 Chaetoceros-genus TARA abundance table The raw data of the abundance of V9 reads of *Chaetoceros* in each sampling locality.

10.7717/peerj.7410/supp-3 Table S3 Chaetoceros-species OSD abundance table The raw data of the abundance of V4 reads of *Chaetoceros* species in each sampling locality.

10.7717/peerj.7410/supp-4 Table S4 Chaetoceros-species TARA abundance table The raw data of the abundance of V9 reads of *Chaetoceros* species in each sampling locality.

10.7717/peerj.7410/supp-5 Table S5 List of taxonomically accepted Chaetoceros taxa according to AlgaeBase Accessed on 15 October 2018.

10.7717/peerj.7410/supp-6 Table S6 List of datasets included in GBIF and OBIS platforms ”GBIF datasets” and ”OBIS datasets” include the names of the datasets in GBIF and OBIS respectively, in which *Chaetoceros* occurence data where gathered. ”unique datasets” includes the list of unique datasets among the two sources, whilst ”shared datasets” lists the datasets included in both platforms.

10.7717/peerj.7410/supp-7 Table S7 List of bibliographic references reporting Chaetoceros at given localities The results of the literature search conducted using Google Scholar ordered by locality.

10.7717/peerj.7410/supp-8 Table S8 Clustering test at 100 and 99% of similarity for V4 and V9 fragments Yellow: highlighted cases in which different species have collapsed together at the set threshold.

10.7717/peerj.7410/supp-9 Table S9 List of OSD and TARA stations Stations in which Chaetoceros samples were not found are highlighted in red. OSD and TARA stations are listed in ”OSD stations” and ”TARA stations” respectively.

10.7717/peerj.7410/supp-10 Table S10 Raw data utilised for plotting maps of *Chaetoceros* species “species for maps” illustrates the way strains were merged to plot species maps; “OSD_coord” includes the OSD Chaetoceros-species abundance table used to plot OSD stations; ”TARA_coord” includes the TARA Chaetoceros-species abundance table used to plot TARA stations.

10.7717/peerj.7410/supp-11 Article S1 Distribution maps of *Chaetoceros* species using OSD and TARA datasets Blue dots refer to occurrences in OSD stations, red dots to occurrences in TARA stations.

## Additional Information and Declarations

### Data Availability

The following information was supplied regarding data availability: The raw data are provided in the Supplemental Files.
