# Supplementary material for: Global distribution and diversity of Chaetoceros (Bacillariophyta, Mediophyceae): integration of classical and novel strategies
Source: PeerJ. 2019 Aug 19;7:e7410. doi: 10.7717/peerj.7410 (PMC6705385; doi:10.7717/peerj.7410)

## Article S1. Distribution maps of *Chaetoceros* species using OSD and Tara Oceans datasets.

OSD (blue dots) and Tara Oceans (red dots) stations.

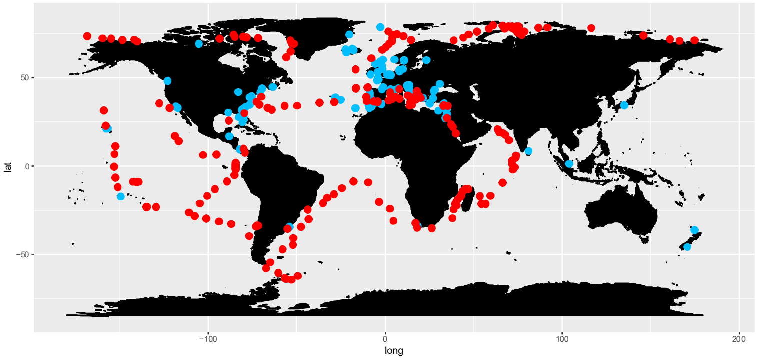


*C. affinis*

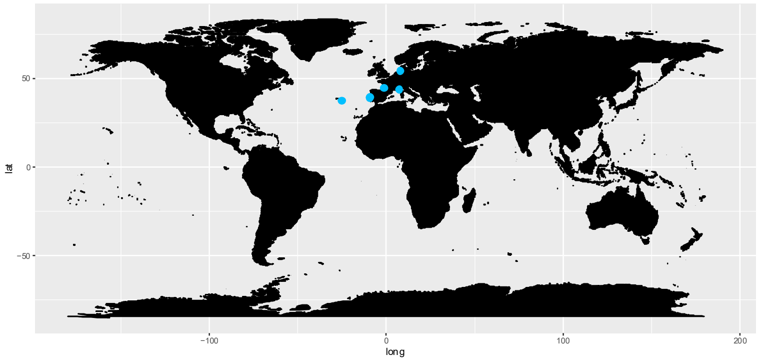


*C. anastomosans*

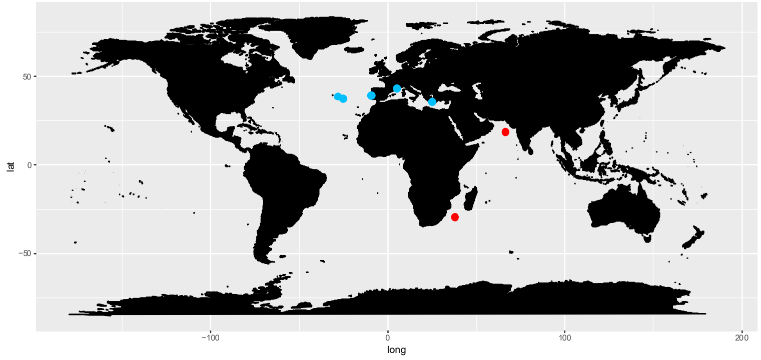


*C. atlanticus*

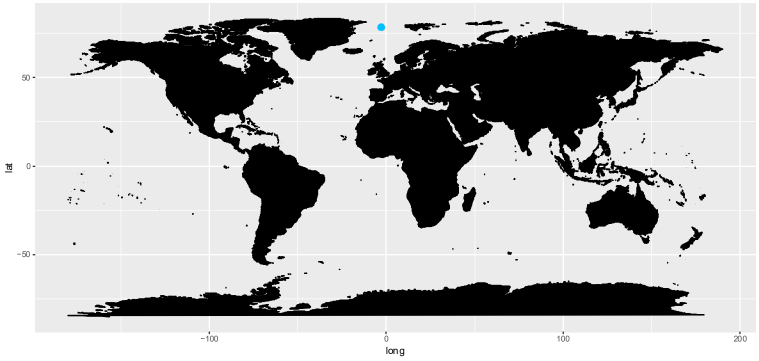


*C. brevis* 1

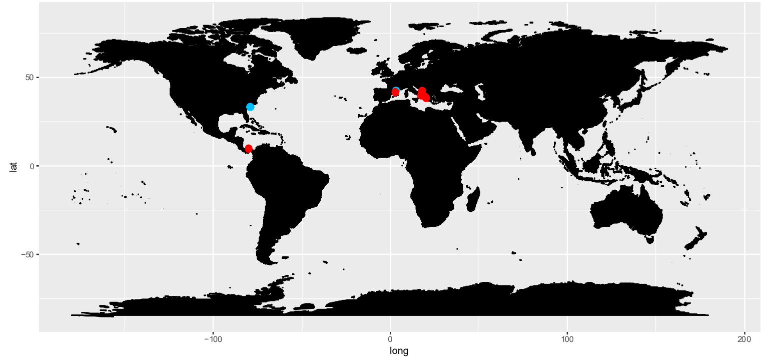


*C. brevis* 2

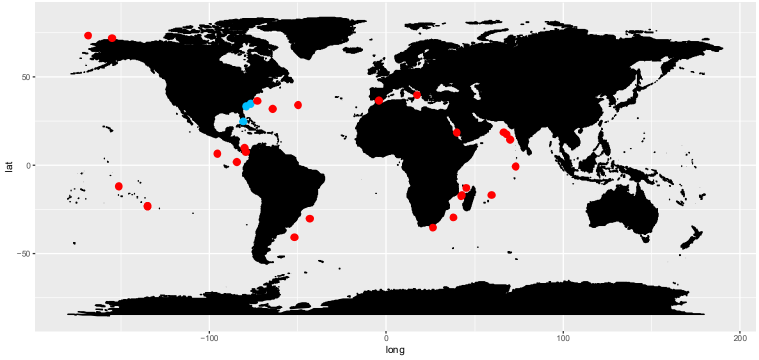


*C. brevis* 3

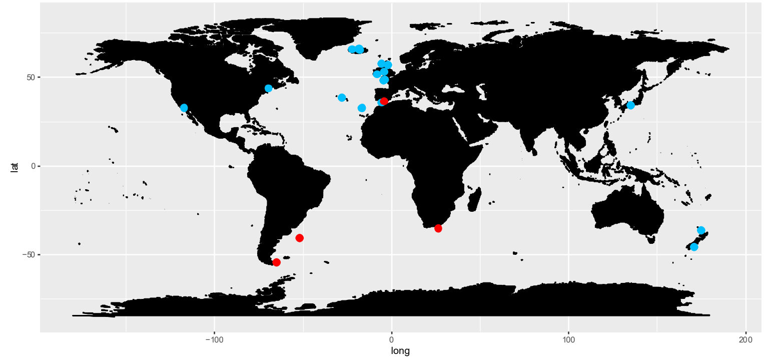


*C.* cf. *convolutus*

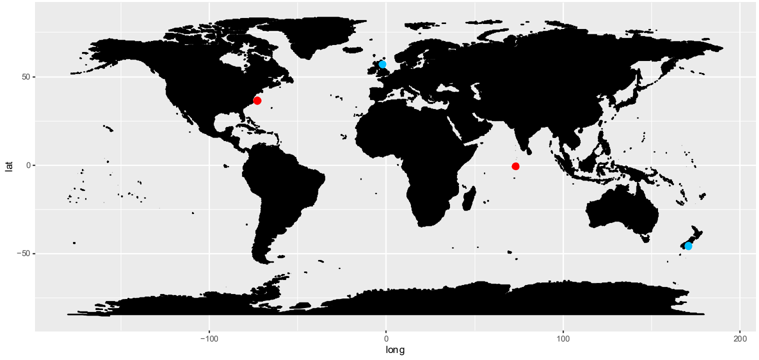

*C.* cf. *pseudodichaeta*

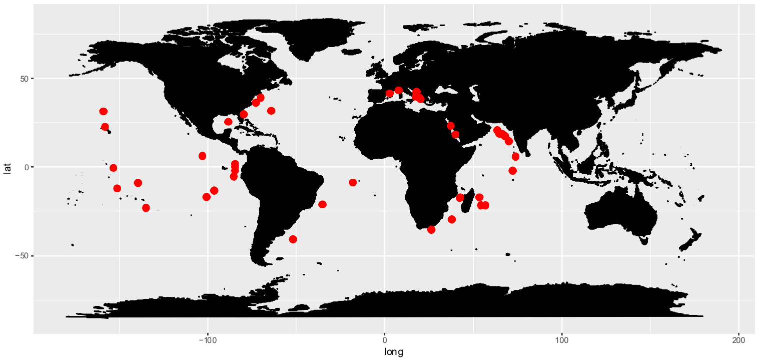


*C.* cf. *tortissimus*

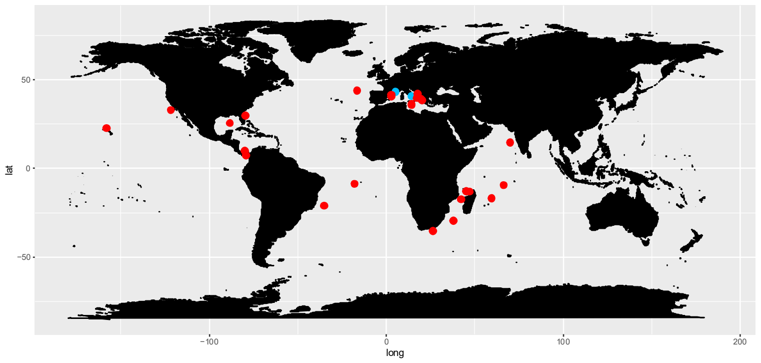


*C.* cf. *vixvisibilis*

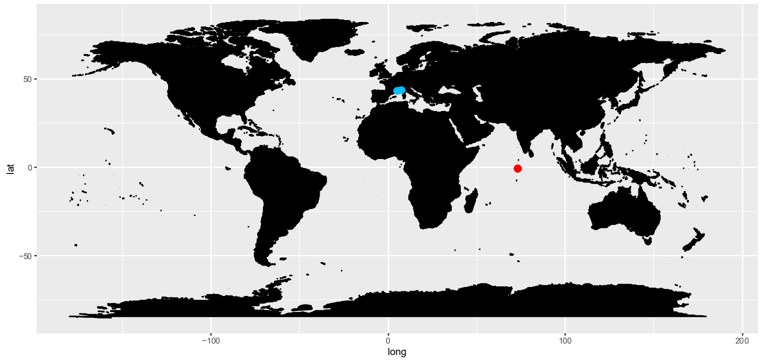


Note: Tara Oceans distribution (red dot) might refer to *C.* sp. Na28A1 due to identical reference sequences in Tara Oceans dataset.

*C. cinctus*

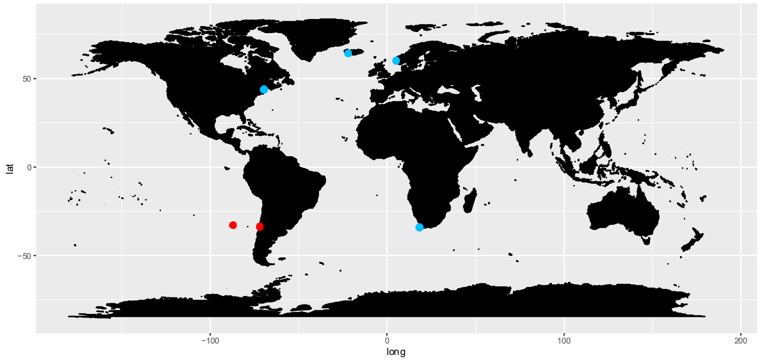


*C. circinalis*

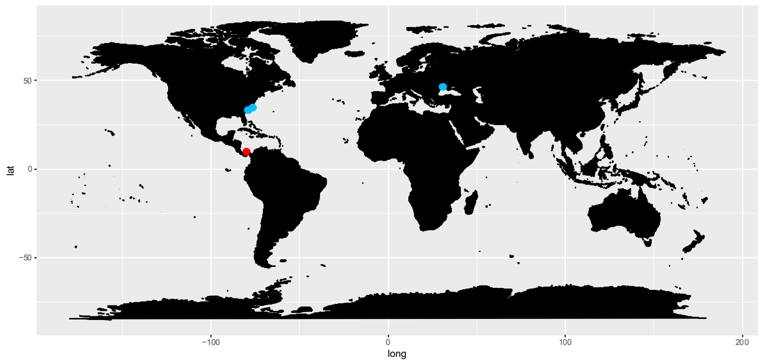


*C. constrictus*

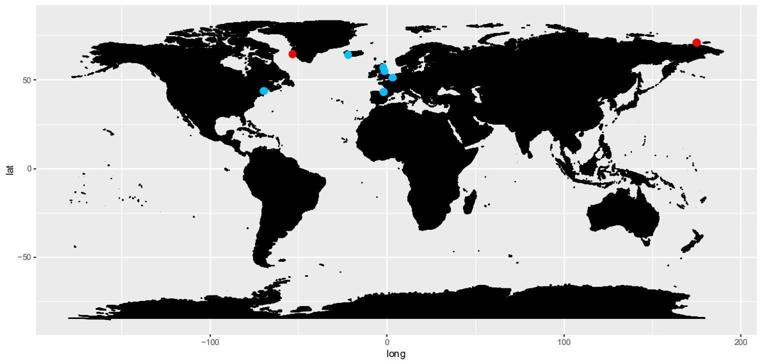


*C. contortus*

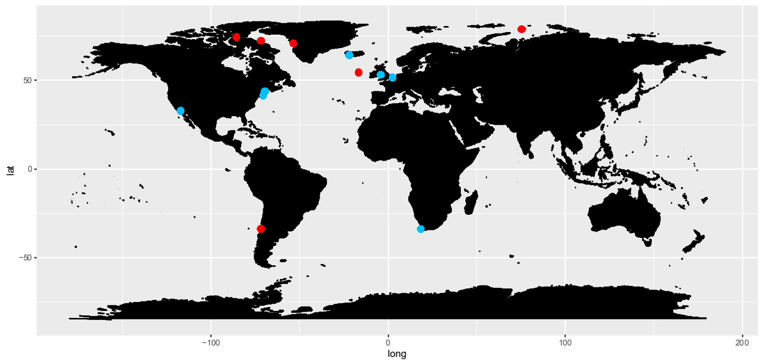


*C. contortus* cf. var. *contortus*

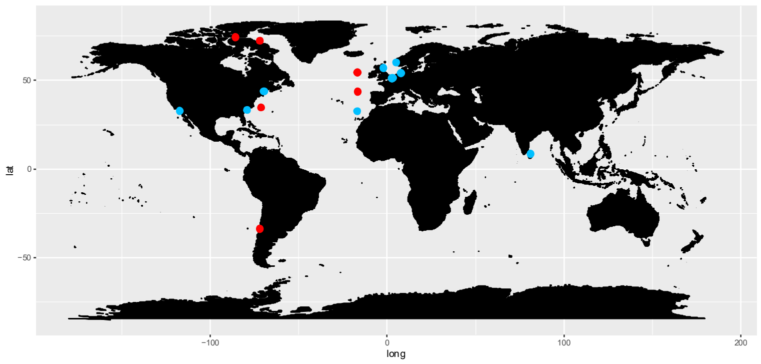

*C. costatus*

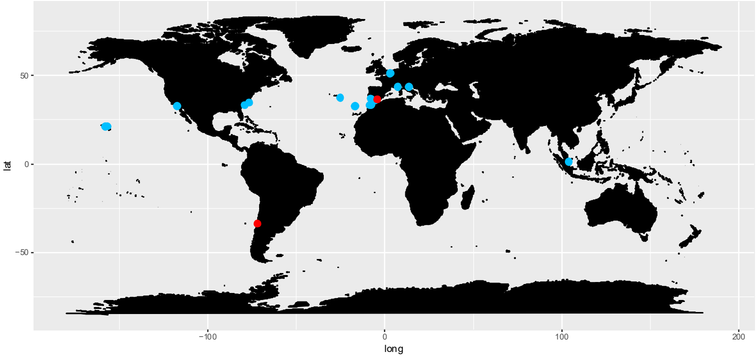


*C. curvisetus* 1

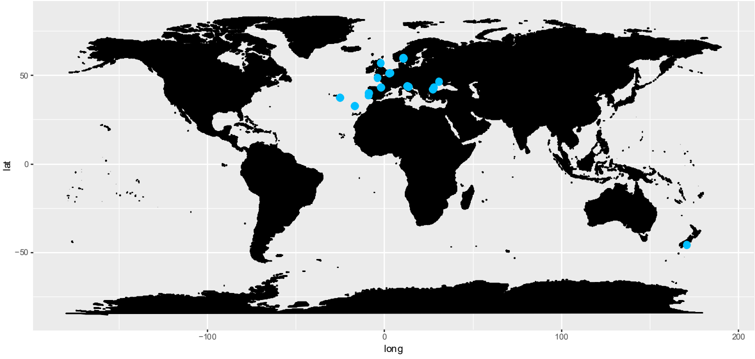


*C. curvisetus* 2

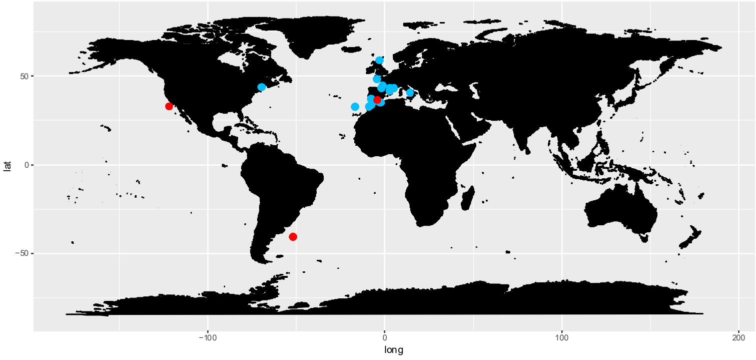


*C. curvisetus* 3

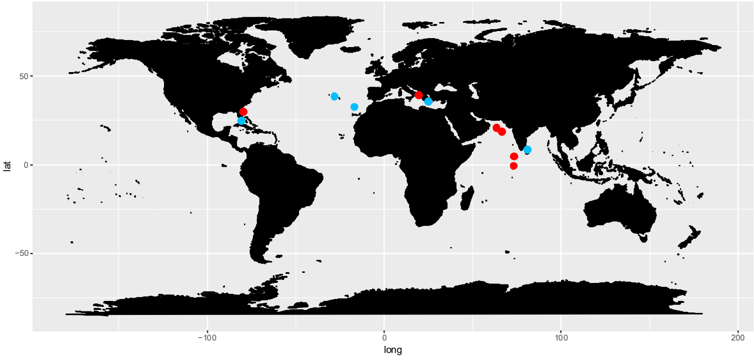


*C. danicus* (strain newCB1)

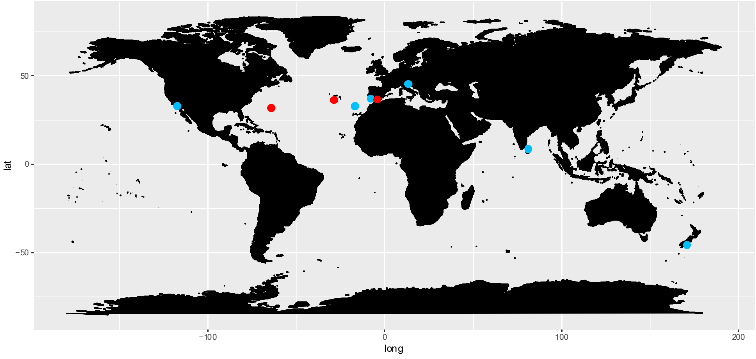


*C. danicus* (strain RCC2565)

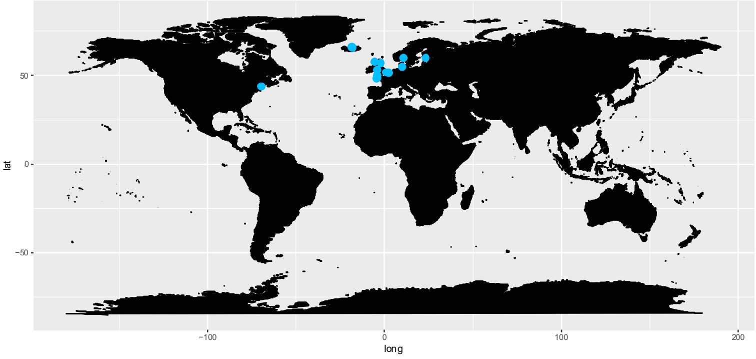


*C. debilis* 1

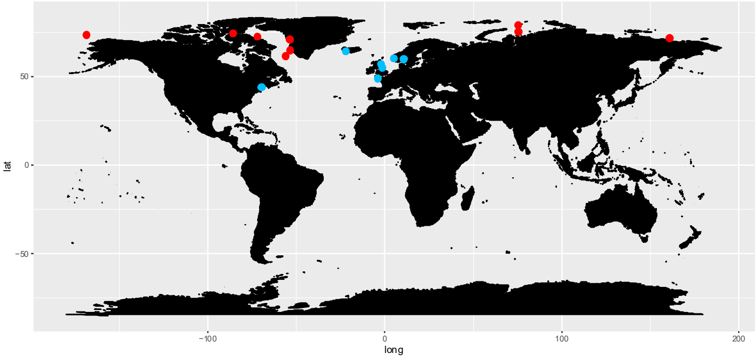


*C. debilis* 2 (strain L38-A2)

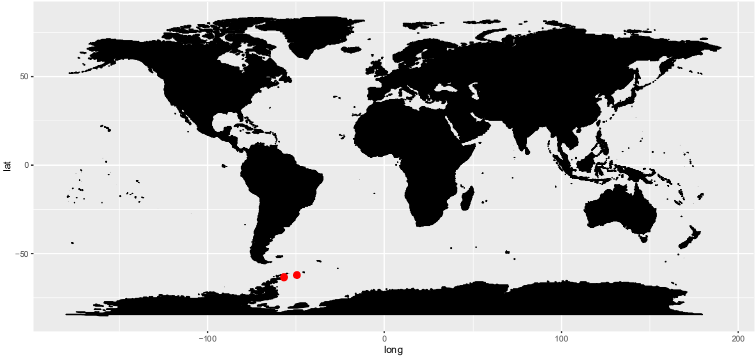

*C. debilis* 2 (strain MM24-A3)

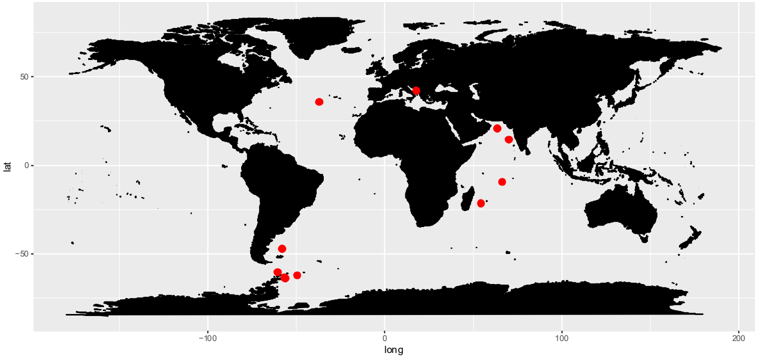


*C. debilis* 3

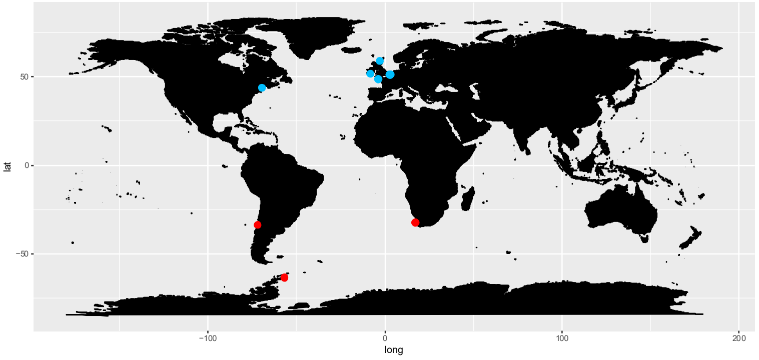


*C. decipiens*

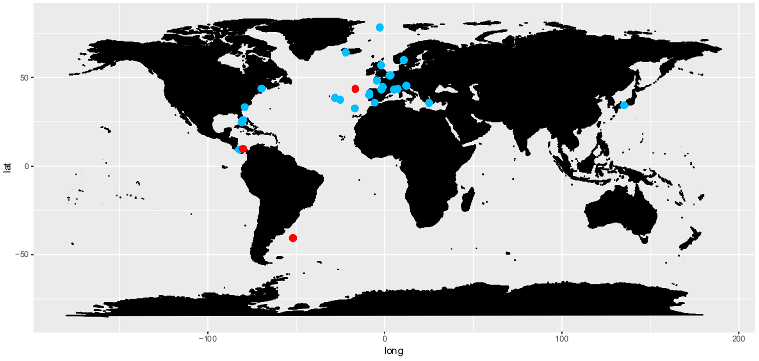


*C. diadema* 1

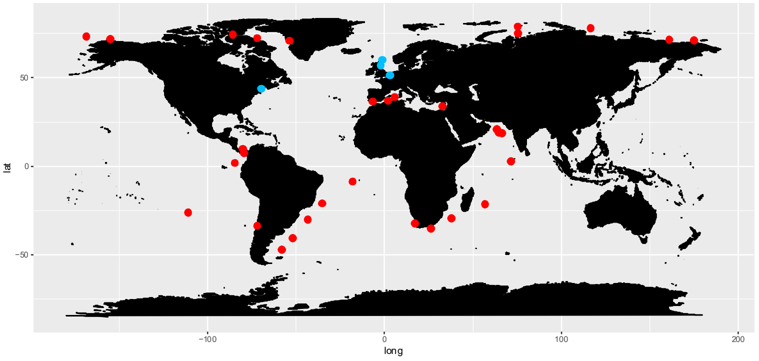


*C. diadema* 2

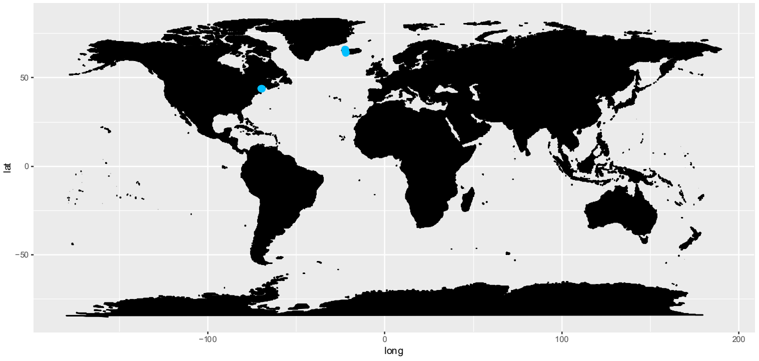


*C. dichaeta*

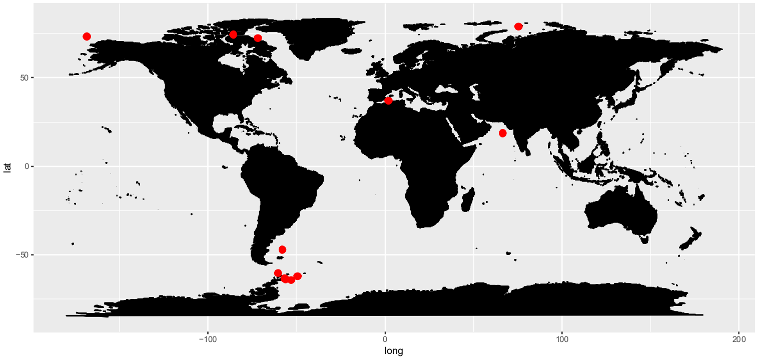


*C. dichatoensis*

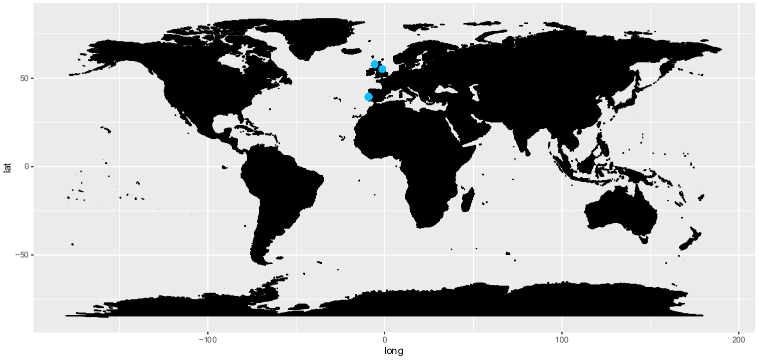


*C. didymus* 1

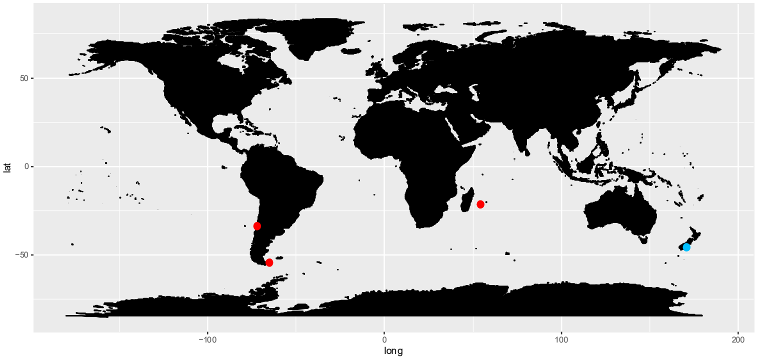

*C. didymus* 2

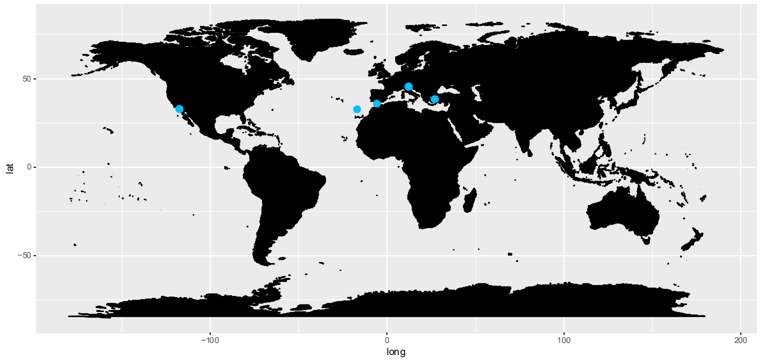


*C. diversus* 1

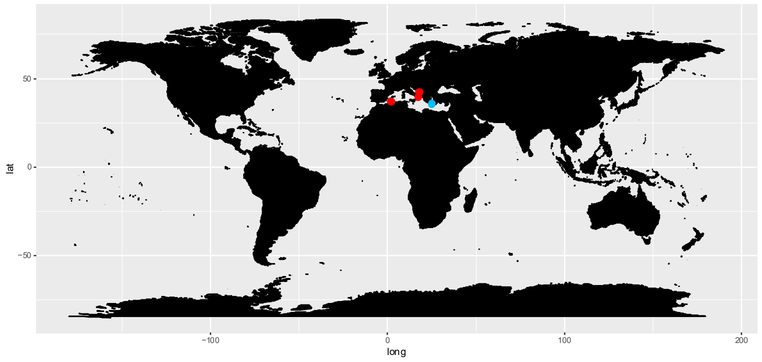


*C. diversus* 2

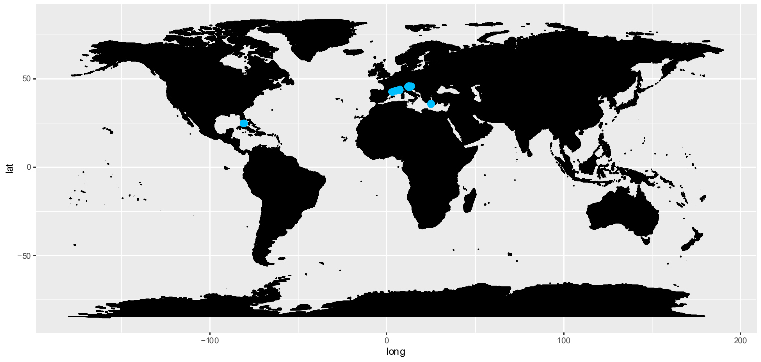


*C. eibenii*

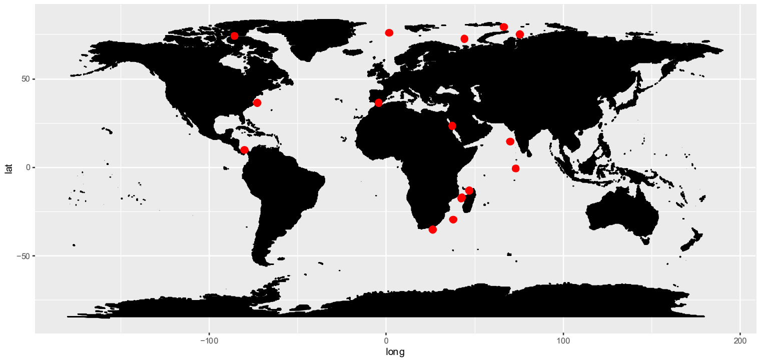


*C. elegans* (strain Ch12A1)

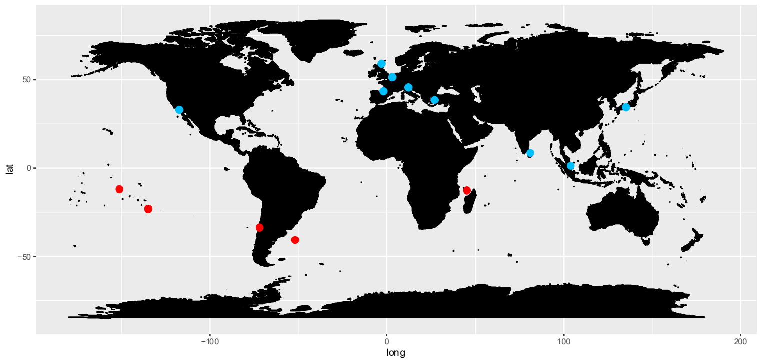


*C. elegans* (strain MC1001)

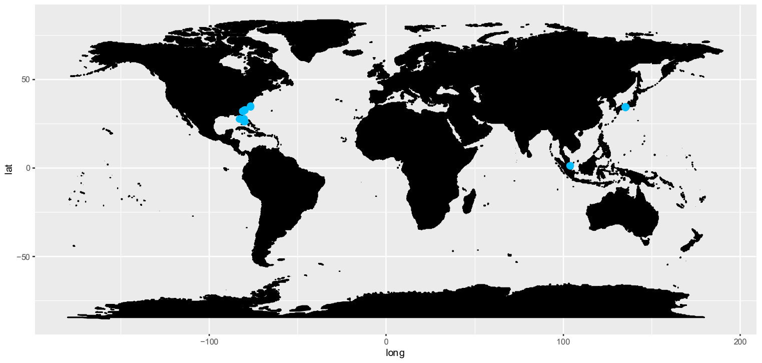


*C. elegans* (strain MC785)

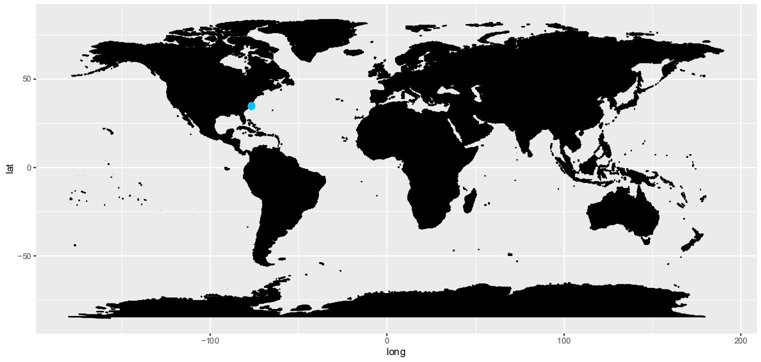


*C. gelidus*

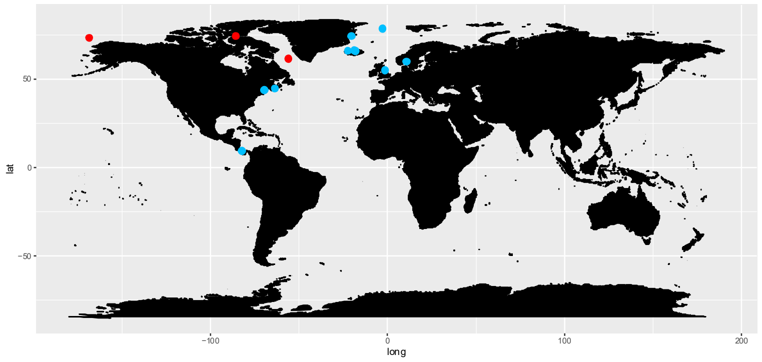

*C. lauderi*

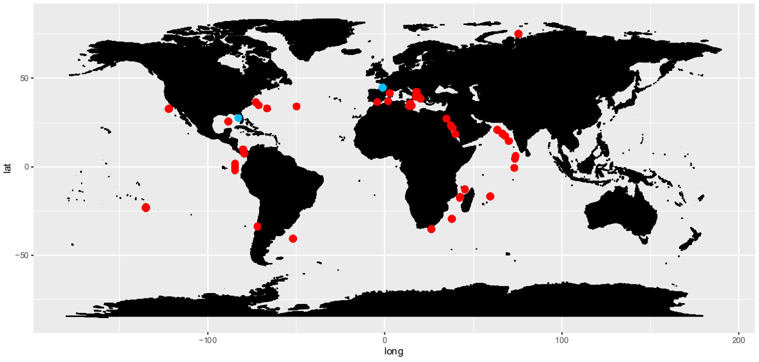


*C. lorenzianus* 1

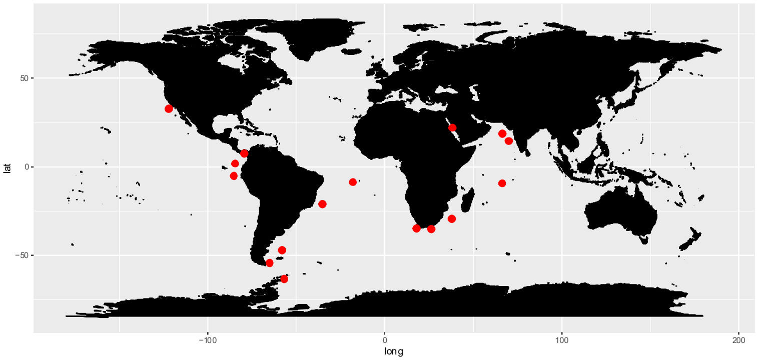


*C. lorenzianus* 2

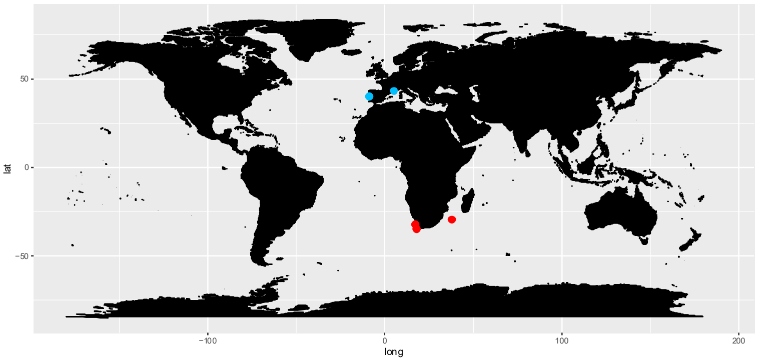


*C. mannaii*

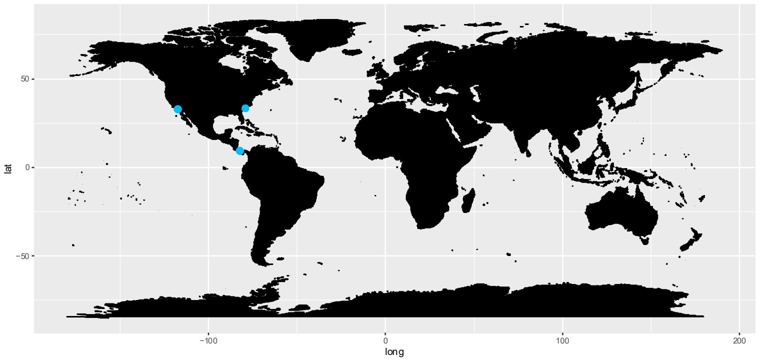


*C. minimus*

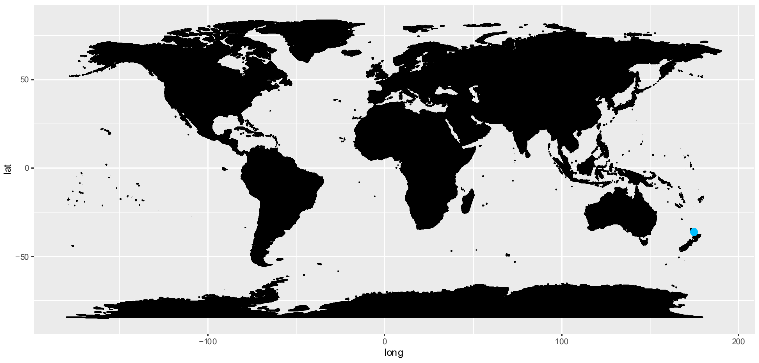


*C. neogracilis*

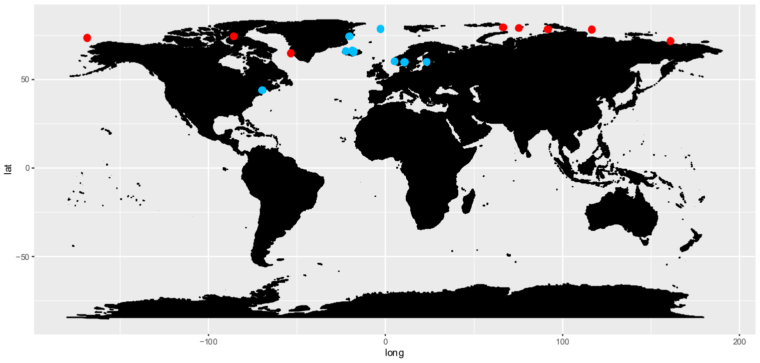


*C. peruvianus* 1

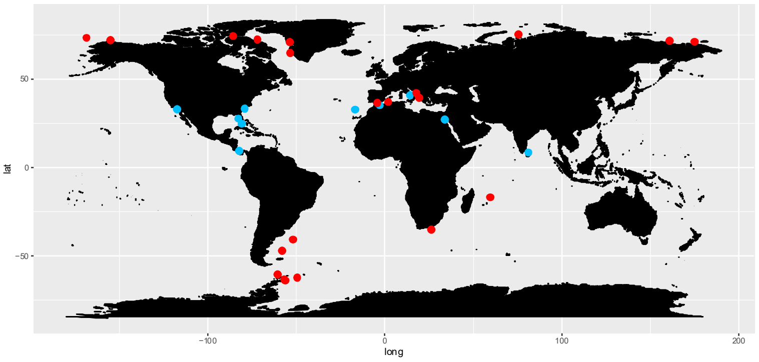


*C. peruvianus* 2

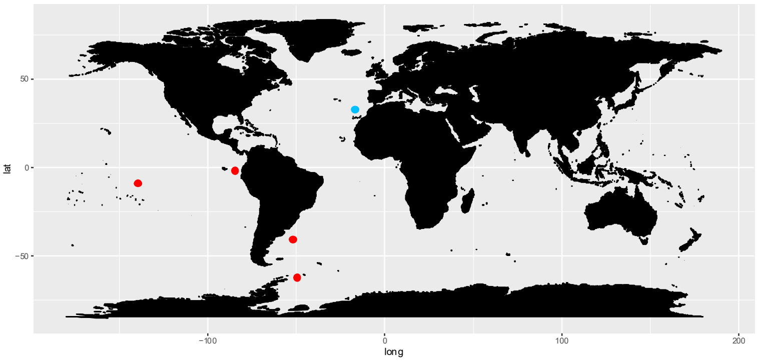

*C. protuberans* (strain Bristol)

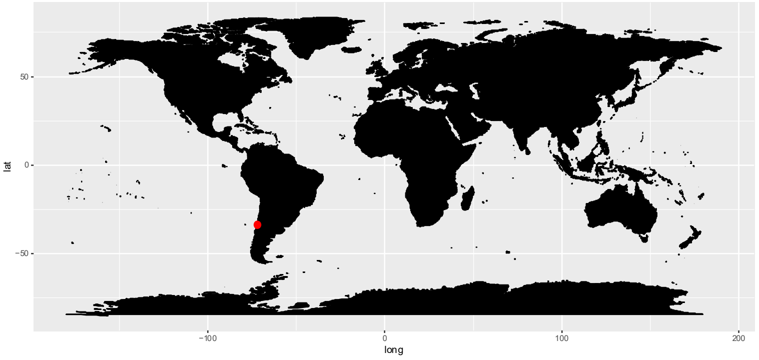


*C. protuberans* (strain newJC4)

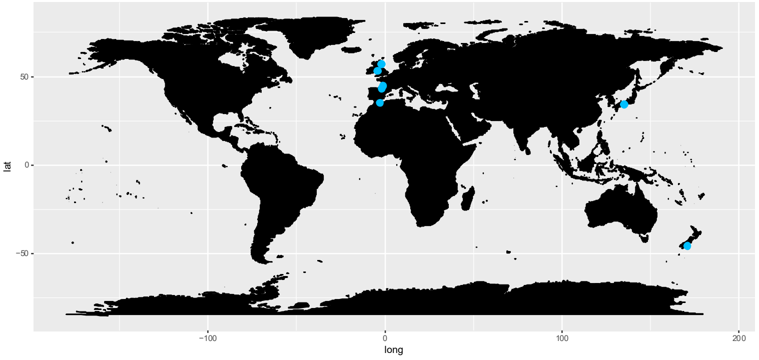


*C. pseudocurvisetus*

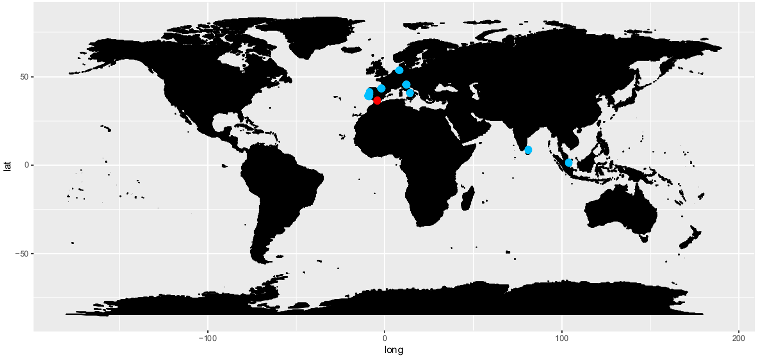


*C. radicans* (strain CCMP197)

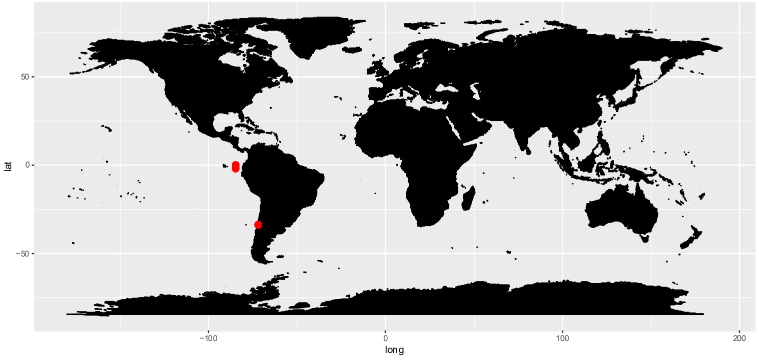


*C. radicans* (strain Ch11A4)

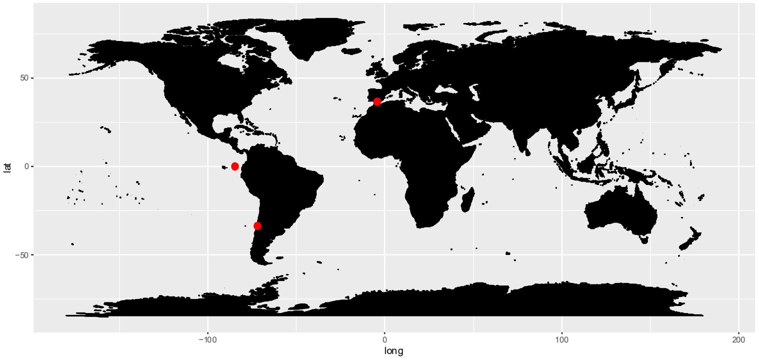


*C. rostratus*

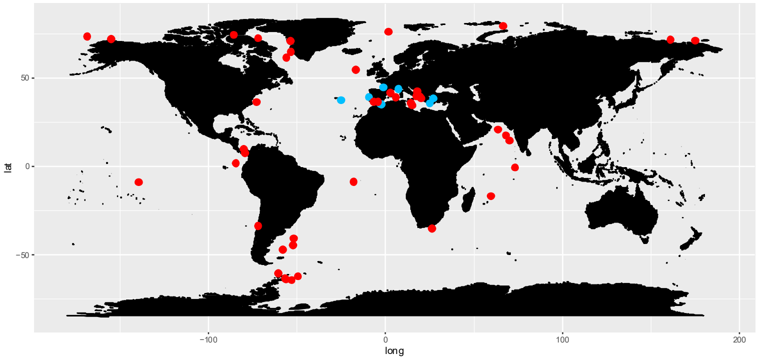


*C. rotosporus*

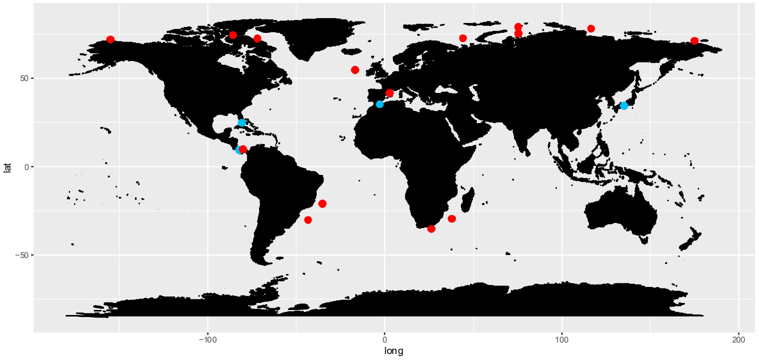


*C. seiracanthus*

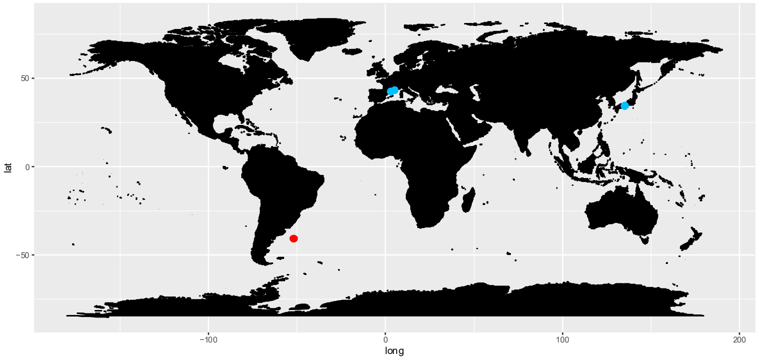

*C. socialis*

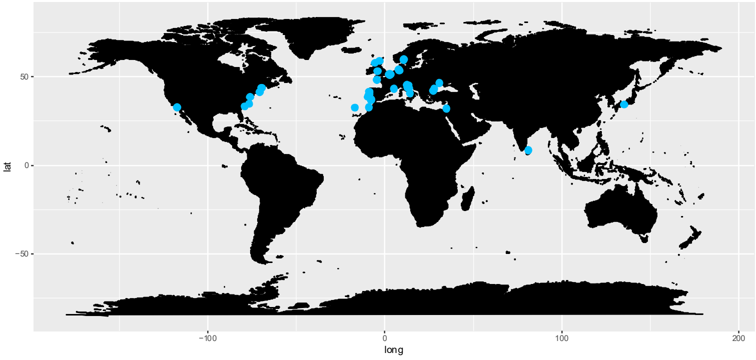


*C.* sp. Clade CDP22

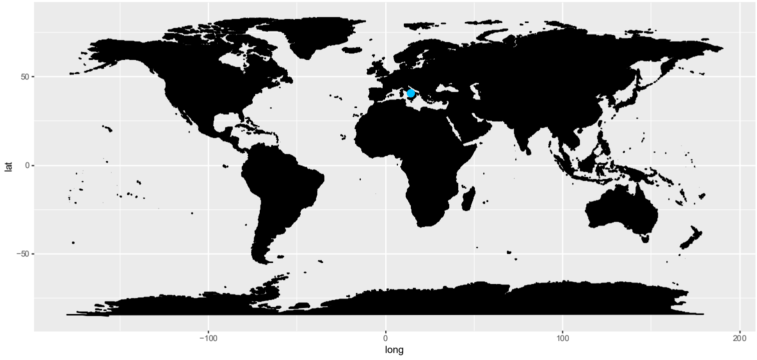


*C.* sp. Clade Na11C3

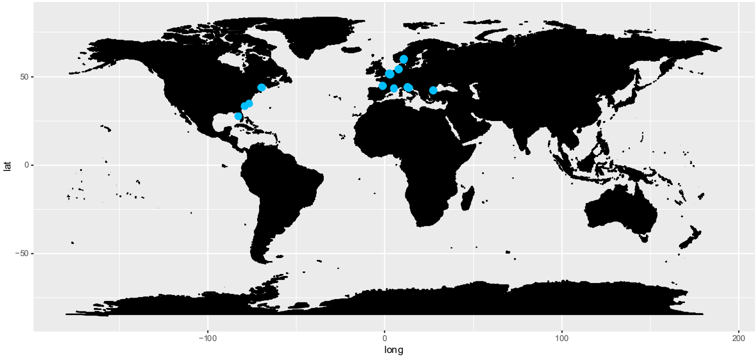


*C.* sp. Clade Na12A3

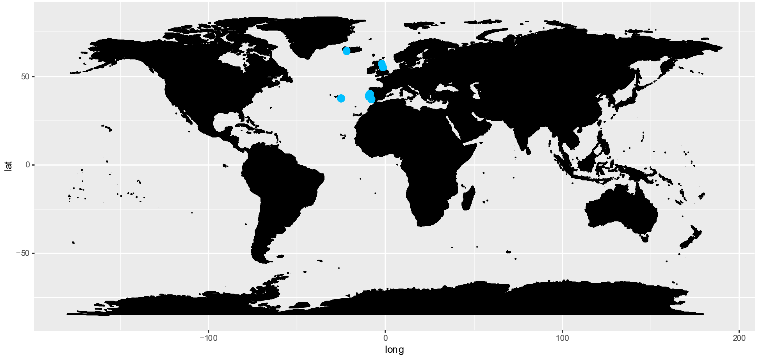


*C.* sp. Clade Na13C1

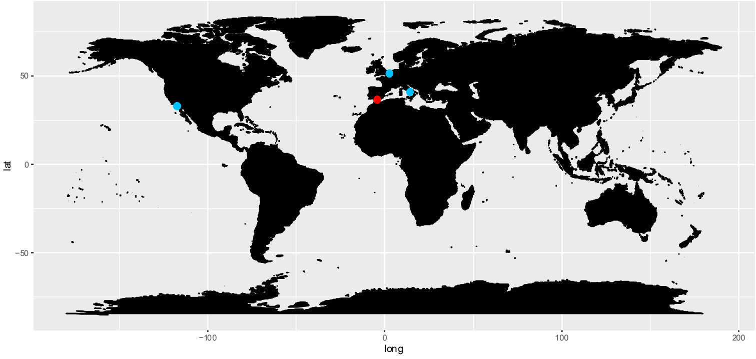


*C.* sp. Clade Na17B2

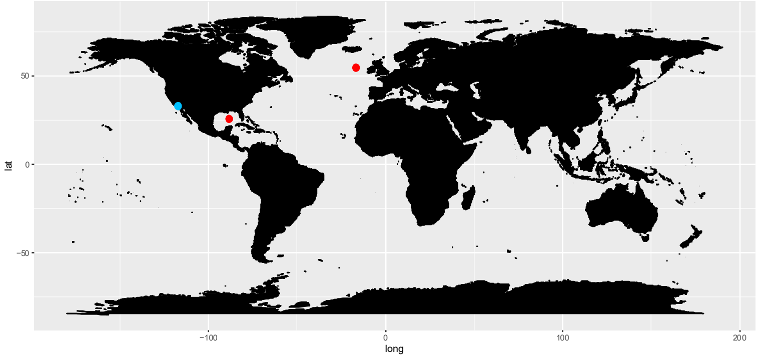


*C.* sp. Clade Na26B1

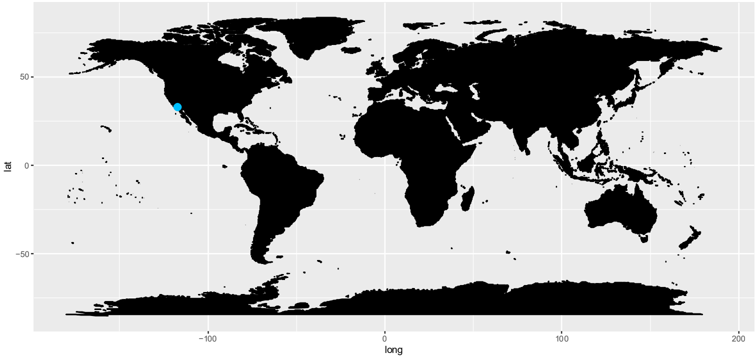


*C.* sp. Clade Na28A1

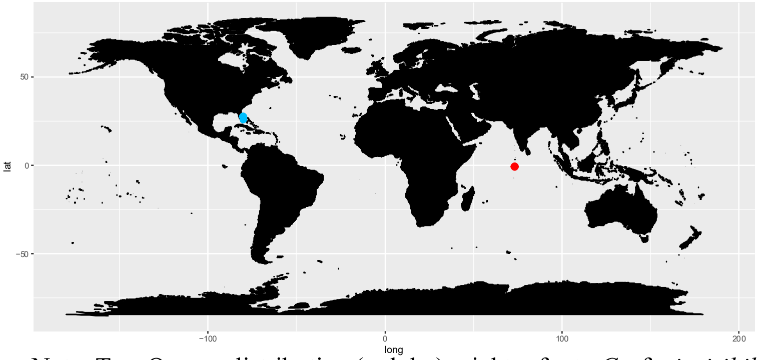


Note: Tara Oceans distribution (red dot) might refer to *C.* cf. *vixvisibilis* due to identical reference sequences in Tara Oceans dataset.

*C.* sp. Clade VA7D2

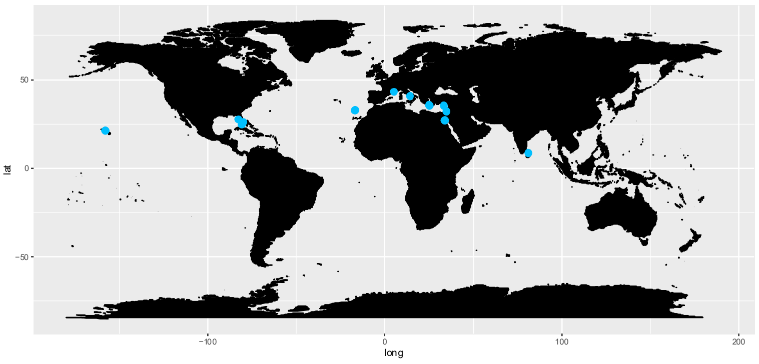


*C. sporotruncatus*

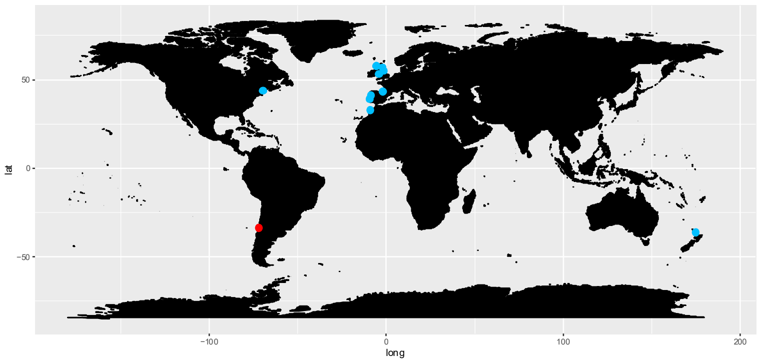


*C. tenuissimus*

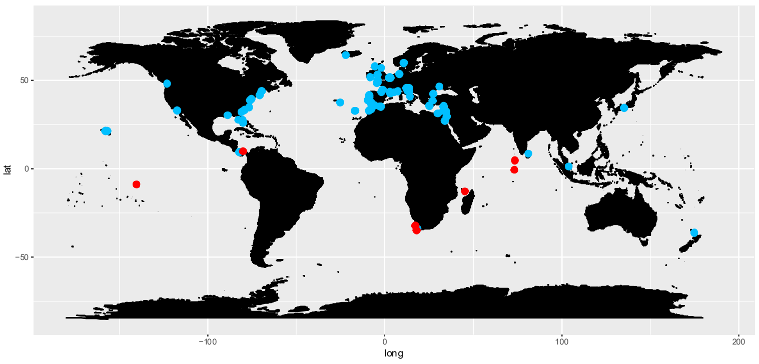


*C. teres*

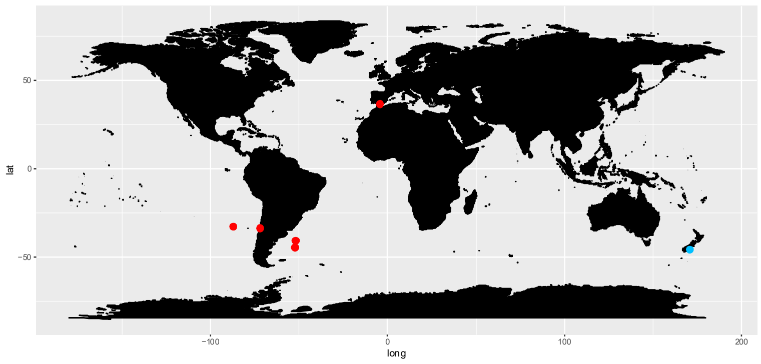


*C. throndsenii*

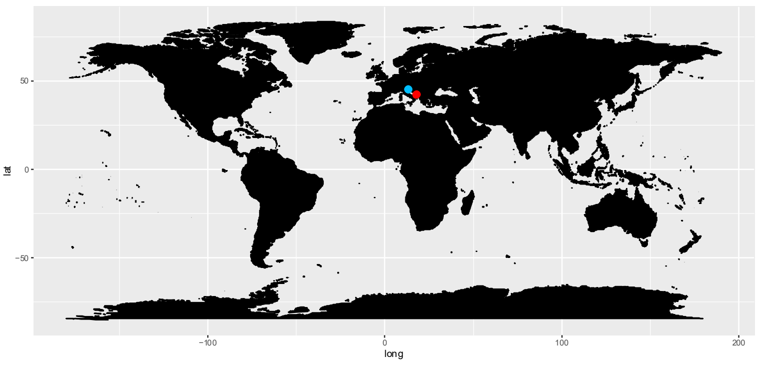


*C. tortissimus*

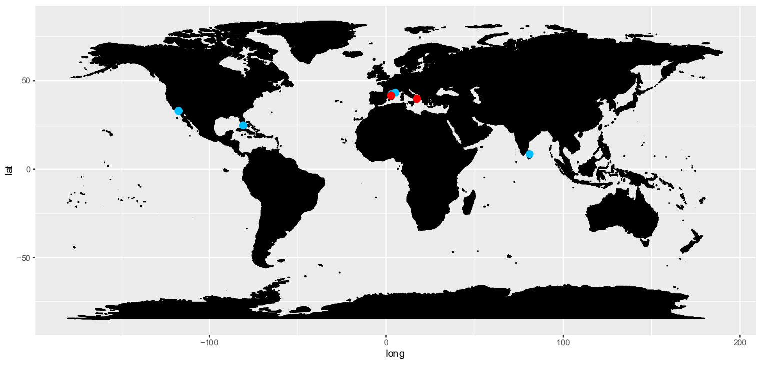

Supplement: Article S1 — Blue dots refer to occurrences in OSD stations, red dots to occurrences in TARA stations. [file peerj-07-7410-s011.pdf]
